# Simulation Addressing Verbal Escalation (SAVE): An Interprofessional Simulation for Pediatric Health Care Professionals

**DOI:** 10.15766/mep_2374-8265.11593

**Published:** 2026-04-15

**Authors:** Abigail Nolan, Mallory Cordes, Ananya Datta, Kellee Humphries, Ayushi Jain, Simmy King, Rosalyn Manuel, Laura Nicholson, Jennifer Owens, Haroon Shaukat, Gregory Yurasek, Pavan Zaveri, Simranjeet S. Sran, Heather Walsh

**Affiliations:** 1 Pediatric Critical Care Medicine Fellow, Children's National Hospital; 2 Simulation Education Specialist, Simulation Program, Children's National Hospital; 3 Simulation Operations Specialist, Simulation Program, Children's National Hospital; 4 Medical Student, The George Washington University School of Medicine and Health Sciences; 5 Director, Nursing Education and Professional Development, Children's National Hospital; Assistant Professor of Pediatrics, The George Washington University School of Medicine and Health Sciences; 6 Executive Director, Clinical Learning and Simulation Skills (CLASS) Center, The George Washington University School of Medicine and Health Sciences; 7 Director of Emergency Department Simulation, Simulation Program, Children's National Hospital; Assistant Professor of Pediatrics & Emergency Medicine, The George Washington University School of Medicine and Health Sciences; 8 Medical Unit Director, Pediatric Cardiac Critical Care Unit; Director of ECMO, Johns Hopkins Children's Center; Associate Professor of Anesthesia and Critical Care Medicine, Johns Hopkins University; 9 Medical Director, Simulation Program, Children's National Hospital; Associate Division Chief for Education, Division of Emergency Medicine, Children's National Hospital; Professor of Pediatrics & Emergency Medicine, The George Washington University School of Medicine and Health Sciences; 10 Director of Education, Simulation Program, Children's National Hospital; Director of Education, Division of Neonatology, Children's National Hospital; Assistant Professor of Pediatrics, The George Washington University School of Medicine and Health Sciences; 11 Simulation Program Manager, Children's National Hospital; Assistant Professor of Pediatrics, The George Washington University School of Medicine and Health Sciences

**Keywords:** Simulation, Verbal Escalation, Standardized Participant, Pediatrics, Communication Skills, Interprofessional Education, Pediatrics, Provider-Patient Relationship

## Abstract

**Introduction:**

Clinicians frequently encounter workplace verbal violence from patients and caregivers. We created Simulation Addressing Verbal Escalation (SAVE) training for hospital-based staff to practice managing verbal escalation in a safe learning environment while reinforcing available hospital resources.

**Methods:**

This curriculum included a 1-hour session with 2 scenarios, each involving interprofessional ad hoc teams simultaneously managing a pediatric patient (manikin) experiencing sepsis with decreased responsiveness, and a verbally escalating caregiver (standardized patient). A debriefing reinforced the BEAR (Bond, Engage, Assess, Reinforce) communication framework, sepsis management, and hospital resources for caregiver support. Training was evaluated using a checklist documenting team actions, and a learner feedback survey (4-point scale; 1 = *nothing/not at all*, 4 = *quite a bit*).

**Results:**

From October 2024 to April 2025, 860 clinicians participated in SAVE training. There were significant increases from scenario 1 to scenario 2 in team implementation of the full BEAR communication tool (35% to 77%, *p* < .0001), the Bond (67% to 94%, *p* < .0001) and Reinforce (56% to 83%, *p* < .0001) components, and SWIFT (Social Work Intervention for Families and Teams) utilization (1% to 6%, *p* = .014). Among survey respondents (*n* = 610), mean ratings were 3.55/4, 3.73/4, and 3.88/4 for learning, engagement, and facilitator effectiveness, respectively. Relevant takeaways included supporting families, considering patient care team dynamics, applying communication strategies, and accessing hospital resources.

**Discussion:**

SAVE training provided a meaningful experience for practice and discussion about emotional clinical situations. Hospital-wide training empowers staff to leverage resources to support patients’ caregivers.

## Educational Objectives

By the end of this activity, learners will be able to:

1. Escalate care of patients presenting with sepsis.
2. Apply BEAR (Bond, Engage, Assess, Reinforce) framework to address verbal escalation by a caregiver.
3. Utilize appropriate resources available locally for behavioral escalation events.

## Introduction

Clinicians encounter verbal and physical violence from patients and their caregivers while providing patient care. Unchecked, verbal escalation can lead to physical threats and ultimately physical violence, posing danger to staff, patients, families, and visitors. Effective de-escalation techniques are crucial in health care settings, particularly within hospitals where staff routinely encounter high-stress situations that can escalate into aggressive or violent behavior.^1–3^ Health care staff experience high rates of violence, with approximately 50% of hospital workers encountering aggressive incidents during their careers and over 70% of all workplace assaults occurring in health care and social services settings.^4,5^ The ability of health care professionals to manage these situations ensures the safety and well-being of patients and staff while fostering a supportive therapeutic environment. Training hospital staff in de-escalation has been linked to reductions in workplace violence and improved staff confidence and safety.^6^ Traditional training methods like role-playing or didactic learning have proven valuable but do not capture the complexity and immediacy of real-life scenarios. As patient aggression rises in hospitals, there is an urgent need for effective and scalable training solutions.

Simulation-based training has emerged as a powerful tool to bridge this gap and allows for the safe rehearsal of de-escalation strategies by providing the opportunity to practice and refine responses in realistic, low-risk settings.^5,7^ However, most existing training programs focus on adult populations in areas such as the emergency room or psychiatric floor. Several curricula exist for medical students^8–11^ and residents^9,12^ focused on verbal de-escalation. Pediatrics involves managing both caregiver and child during escalations, each requiring distinct skills. To meet this challenge, we created Simulation Addressing Verbal Escalation (SAVE) training to help clinicians use verbal de-escalation techniques and hospital resources for supporting parents and staff. To our knowledge, this is the first hospital-wide simulation-based training addressing verbal escalation and clinical deterioration intended for pediatric interprofessional clinicians.

## Methods

### Development

Our interprofessional simulation team, led by a nurse and physician educator, developed SAVE training using Kern's six-step approach.^13^ Step 1 included a general needs assessment, which was conducted through organizational trends on verbal violence and leadership requests followed by a targeted needs assessment of the intended audience through stakeholder engagement (step 2). Our team outlined goals and objectives (step 3), created the content (step 4), implemented the curriculum (step 5), and evaluated the curriculum (step 6). We note specific steps used in the curriculum design, implementation, and evaluation below. SAVE is an interprofessional educational activity where learners focus on providing care in acute situations while leveraging their skills to communicate effectively with a distraught caregiver. Simulation nurse and physician leaders met with senior hospital leaders including the chief nursing officer, chief quality and safety officer, chief clinical officer, and vice-chair of medical education and key stakeholders and joined relevant committees to garner support for this initiative. We identified educational objectives (step 3), and created a 1-hour required simulation-based session for all nurses, physicians, advanced practice providers, technicians, unit support associates, social workers, respiratory therapists, unit clerical associates, child life specialists, pharmacists, and others with patient-facing roles. Continuing education was offered for nurses, physicians, advanced practice providers, social workers, and pharmacists. The Children's National Hospital Institutional Review Board approved this project (No. MOD00008483; June 17, 2025). Participant evaluation focused on immediate learning.

The curriculum consisted of 2 simulation scenarios (step 4), each with a decompensating patient (high-fidelity manikin) (Appendix A) accompanied by a caregiver portrayed by a trained standardized patient (SP) (Appendix B). We decided the SP should portray the caregiver to ensure proper escalation. We selected the clinical scenario of sepsis due to its relevance across specialties and the difficulty providers face in timely identification and management. Both scenarios occurred during shift change, when the incoming nurse received minimal hand-off and a caregiver voiced concern in the hallway about their child. The second scenario happened a year later when the patient was hospitalized again, and the caregiver was highly distressed. In both scenarios, participants were expected to assign roles, manage a patient with septic shock, and engage with the caregiver to identify and validate concerns.

The participating SPs were recruited and trained for the simulation following the Association of SP Educators (ASPE) Standards of Best Practice (SOBP).^14^ The ASPE SOBP identify core values for working with SPs including maintaining a safe environment, collaboration between SPs and simulation stakeholders, professionalism, and quality. The SP training was conducted with an SP Educator and the lead nurse and physician educator from the simulation team. SPs were trained to portray the same caregiver in both scenarios.

We created a learner guide (Appendix C) reinforcing evidence-based sepsis guidelines and hospital resources for clinical and verbal de-escalation. This guide incorporated resources, including resources for verbal de-escalation, into a single reference source, which is posted on the hospital's intranet site. We deemed it important to provide a framework to guide staff communication with caregivers and families. In engaging with stakeholders, we encountered the BEAR (Bond, Engage, Assess, Reinforce) framework for communication, which was created by our hospital patient experience team in 2022, with dissemination limited to new hires. We decided to adopt this communication framework and reinforce its use in interacting with patients and families. The learner guide provided scripting and strategies for handling families showing distress and reminded staff to stay calm and use open body language.

Each session could accommodate 4 physicians or advanced practice providers, 6 nurses, and 4 others in clinical roles (e.g., respiratory therapists, social workers, patient care technicians). If there were no physicians, advanced practice providers, or nurses in a session, 1 of the simulation educators assumed that role to advance the scenario, allowing the other learners to support the caregiver (the SP). There was no required prework, though the learner guide was attached to the training appointment. Each session was 1 hour in length, with 10 minutes allotted for each simulation and 15 minutes for the debrief after each case (details on how we developed the curriculum content [step 4] are provided below). We determined simulation to be the most effective teaching strategy to meet the educational objectives, and we developed a facilitator guide (Appendix D) to standardize the curriculum across many sessions.

The training began with a standard prebriefing and introductions, where participants shared family stressors to generate conversation and draw on their experiences. To maximize learning, the scenarios were designed to increase in complexity. We reviewed the training slides (Appendix E) during the prebriefing and the debriefing, to display ground rules, sepsis guidelines, escalation resources, and the BEAR communication framework, allowing participants to apply them during the second case.

The BEAR framework, created by our hospital's patient experience team in 2022, has not been validated. We decided to use this model, instead of adopting an alternative framework, because it is already being used in our organization. In the BEAR framework, bonding (B) with patients and families includes establishing a connection through introductions, considering body language, and assessing caregiver well-being (e.g., offering tissues, food or drink, a blanket, etc.); interventions to engage (E) with families include building a partnership, using active listening, asking questions, and validating feelings; staff can assess (A) by evaluating the partnership with caregivers, clarifying understanding of the situation, and managing expectations, through self-reflection and providing frequent check-ins; steps to reinforce (R) communication with families include summarizing interactions, validating caregiver perspectives, and ensuring they have the information necessary for next steps (e.g., plan of care).

Depending on class size, either 1 small team participated in both sessions or the group was divided into 2 equal teams. Participants assumed their typical roles, though there was some limitation for individuals who do not typically engage in patient care (e.g., unit clerks). These individuals often engaged with the parent in a support role with nurses and social workers. Roles were not preassigned other than providers and nurses, which allowed teams to organically assign roles, whether managing caregiver escalation or obtaining patient supplies or snacks for the caregiver. With larger classes, we asked observers to provide peer feedback in the debriefing, and asked simulation participants to reflect on their performance, including what went well and opportunities for improvement. SPs delivered feedback during the debrief, focusing on the healthcare team's communication and overall interactions in the scenarios.

### Equipment/ Environment

We used a high-fidelity toddler manikin for simulations reflecting clinical settings (i.e., acute care floor or emergency department). A patient bed/crib, monitor, and other supplies simulated a realistic clinical setting (see technical support checklist in Appendix F). The set-up can be adapted to any available space, and in low-resource settings, a low-fidelity manikin can serve as the patient, as the primary focus was verbal de-escalation of the caregiver. In settings without SPs, simulation staff or educators can portray the caregiver.

### Personnel

Each session required 2 facilitators to lead the discussion and debriefing and 1 simulation operations specialist to run the manikin and reset equipment. In both scenarios, the bedside nurse and team were given a brief handoff, with the ad hoc^15^ team coming to assist when they heard commotion in the hallway.

Facilitators included the core simulation team, simulation leaders, patient experience staff, the ombudsman (patient advocate), unit-based nurse educators, and physician educators who facilitated the simulation. One SP portrayed the caregiver in each scenario and appropriately de-escalated depending on participant tactics. A few times, due to emergencies or scheduling issues, 2 simulation educators who regularly facilitated the SAVE training portrayed the role of SP. In these instances, educators closely replicated the trained SP's actions and behaviors. Occasionally, a simulation educator needed to portray the provider and focused on sepsis management, allowing participants to communicate with the SP.

### Implementation

For curriculum implementation (step 5), the nurse and physician educator created the patient scenarios (Appendix A) and SP scripts (Appendix B). First, we shared the SP scripts with the SP educator and SPs to review before a virtual training/read-through of the scripts. This allowed the SPs to ask clarifying questions and consider any missing data elements. After SP training and scenario pilot, the nurse and physician educators revised the scenarios to improve role/situation depiction and participant safety. The edited scenarios were sent to the SPs and SP educator for review, before train-the-trainer sessions were implemented.

We then held 3-hour train-the-trainer sessions, involving 2 consecutive 90-minute sessions on 2 days. The SPs and the SP educator attended the train-the-trainer sessions, taking turns portraying the SP and observing. We advertised the sessions to key stakeholders interested in serving as facilitators as well as the core simulation team and simulation leaders. The nurse and physician educators led the co-facilitated train-the-trainer sessions, with facilitators serving as scenario participants. Facilitators should possess strong communication skills, the ability to create a safe learning environment, an understanding of de-escalation techniques, simulation facilitation, and debriefing and reflective practice. The training lasted 60 minutes, with 30 minutes allotted for facilitator role discussion and questions. These sessions trialed scenarios with the facilitator guide. After the first train-the-trainer session, we debriefed with the SPs and SP educator, editing the scenario and SP script based on feedback. We repeated this process after the second session, with a few more modifications.

The chief nursing officer and chief quality and safety officer sent an email announcement, along with a training flyer (Appendix G), describing the SAVE training to nurses, physicians, and advanced practice providers. The flyer contained a QR code linked to the signup portal, which accommodated 14 learners per session. Sessions with 3 or fewer learners were canceled.

This is the fourth hospital-wide simulation-based training initiative in our organization since 2016, with implementation guided by previous successes. We offered sessions in 4-hour blocks, either morning or afternoon, during weekday business hours from October 2024 through April 2025. One block of Saturday sessions was offered to accommodate staff strictly working weekends, and weekend night staff attended Monday morning sessions. Night sessions were not provided due to poor historical attendance related to limited staffing. The simulation team tracked attendance via a spreadsheet and regularly shared it with unit/division leaders.

### Debriefing

Debriefing plays a crucial role, mirroring real-life situations involving caregiver verbal escalation. It not only helps participants process their emotions and reflect on their experiences but also equips them with practical tools and strategies for future encounters.^16^ The debrief following each case enabled learners to discuss their initial feelings and receive caregiver-focused feedback from the SP. The first debrief focused on the BEAR communication framework, sepsis management, and clinical de-escalation skills (see Appendix E for slides). The debrief after the second scenario reinforced hospital verbal de-escalation resources and encouraged individual and group reflection on how different caregiver characteristics (e.g., gender, stature, language, experiences with the health care system) might affect the situation, encouraging learners to examine their own biases (see Appendix D for debrief questions).

### Assessment

In our approach to curriculum evaluation (step 6), immediately after the SAVE training session, we asked participants to complete a training feedback survey (Appendix H) to reflect on learning, engagement, facilitator effectiveness, and meaningful takeaways to practice. After completing the postsession evaluation, nurses, physicians, advanced practice providers, social workers, and pharmacists could claim continuing education credit. Facilitators completed a debrief worksheet (Appendix I) following each scenario, capturing data on session logistics (i.e., date, number of learners, interprofessional participation, technical issues, and comments) and on the components of the BEAR communication framework used by teams of learners in each scenario. Facilitators documented how each team, in both simulation scenarios, escalated sepsis care and used hospital de-escalation resources, such as social workers or the Social Work Intervention with Families and Teams (SWIFT) response. SWIFT is an early de-escalation response program focused on parents, guardians, or relatives, ensuring that employees, families, and visitors are always protected and safe. The response team includes a social worker and security, who remain in the background if needed.

We used McNemar's test to compare team performance between scenario 1 and scenario 2, using Microsoft Excel for Microsoft 365 (version 2511, build 19426.20218 Click-to-Run). *P* values ≤.05 were considered statistically significant.

## Results

Between October 2024 and April 2025, 860 interprofessional clinicians participated in SAVE training, including 374 nurses, 167 physicians, 66 advanced practice providers (nurse practitioners, physician assistants), 64 technicians, 36 respiratory therapists, and 18 unit clerical associates, among others. Participant demographic information was collected via the presurvey (Appendix J).

**Figure 1.**
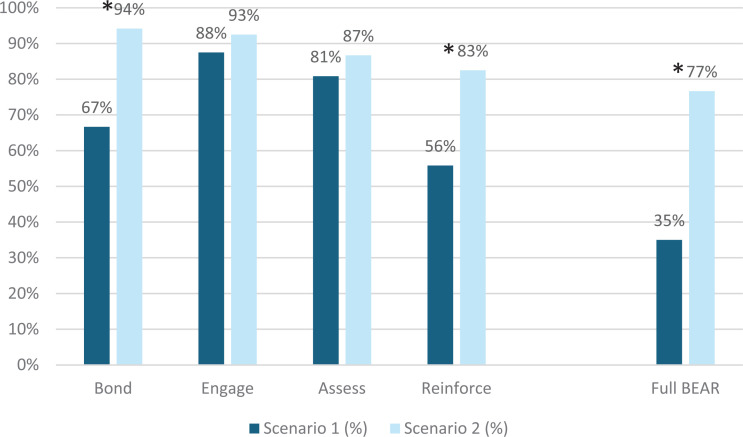
Percentage of participants in the SAVE (Simulation Addressing Verbal Escalation) training scenarios in which teams of learners (*N* = 120) used BEAR framework elements (Bond, Engage, Assess, Reinforce) to communicate with the caregiver (standardized patient). *Indicates statistically significant difference (*p* ≤ .05) between scenario 1 and scenario 2 (determined by McNemar's test).

When performance of the teams (*N* = 120 teams) was compared between scenario 1 and scenario 2, with differences assessed using McNemar's test, implementation of the full BEAR communication tool increased from 35% of participants in scenario 1 to 77% in scenario 2 (*p* < .0001) (Figure 1). Within this framework, use of the Bond communication component increased from 67% to 94% (*p* < .0001), use of the Engage component increased from 88% to 93% (*p* = .109), use of the Assess component increased from 81% to 87% (*p* = .194), and use of the Reinforce component increased from 56% to 83% (*p* < .0001).

**Figure 2.**
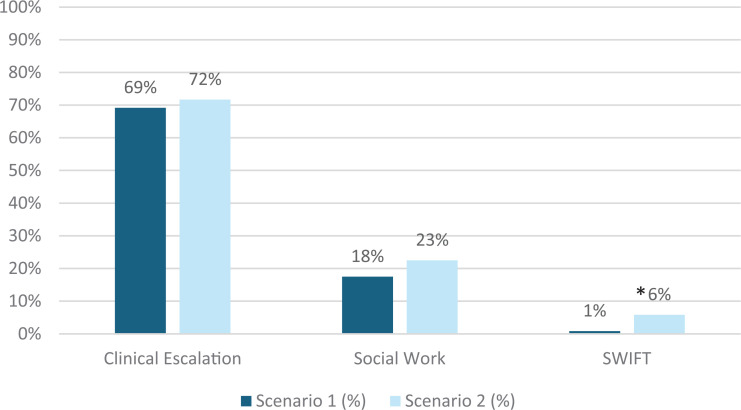
Percentage of participants in the SAVE (Simulation Addressing Verbal Escalation) training scenarios in which teams of learners (*N* = 120) activated clinical escalation, social work, and the SWIFT response (Social Work Intervention for Families and Teams; social worker + security) to escalate sepsis care while de-escalating the caregiver's (standardized patient's) response. *Indicates statistically significant (*p* ≤ .05) difference between scenario 1 and scenario 2 (determined by McNemar's test).

From scenario 1 to scenario 2, use of clinical escalation tools (code blue, rapid response, medical alert) for management of a patient with sepsis increased from 69% to 72% of participants (*p* = .602) (Figure 2). Utilization of social work resources increased from 18% to 23% (*p* = .289), and use of SWIFT de-escalation techniques increased from 1% to 6% (*p* = .014) (Figure 2).

Of 860 participants, 611 completed the training feedback survey, in which they used a 4-point Likert scale (1 = *nothing/not at all*, 4 = *quite a bit*) to rate post-session changes in learning, engagement, and facilitator effectiveness. The mean scores for learning, engagement, and facilitator effectiveness were 3.55/4 (*n* = 610), 3.73/4 (*n* = 610), and 3.88/4 (*n* = 611).

Participants shared key takeaways (Table 1), which included training feedback, importance of family support, impact of patient care team dynamics, use of verbal and physical communication strategies, and available hospital resources. Further, they highlighted what went well in this educational activity (Table 2), which included SP portraying the parent, interprofessional teams, and general feedback on the scenarios and facilitation.

**Table 1.**
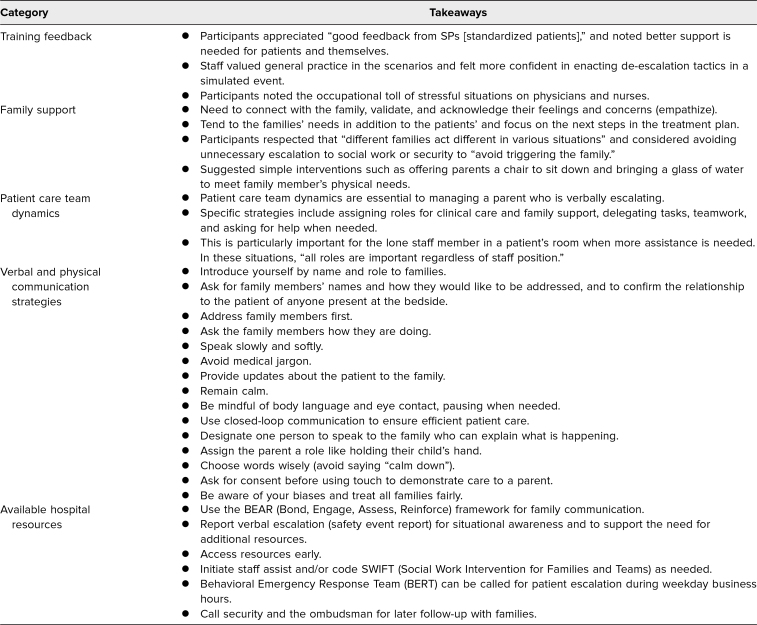
Summary of Participant Takeaways From the SAVE (Simulation Addressing Verbal Escalation) Training (*N* = 611)

**Table 2.**
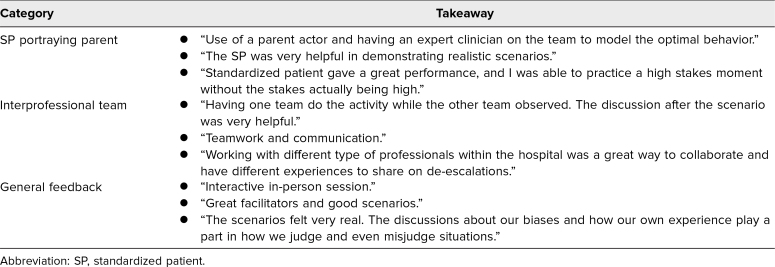
Summary of Participant Feedback on What Went Well in the SAVE (Simulation Addressing Verbal Escalation) Training (*N* = 611)

In addition, participants shared recommendations for improving the activity. These included preference for smaller groups to allow for more participation, potentially making the verbal de-escalation more challenging (refusing care), and guidance on what to do when you feel unsafe and need to leave. Requests included additional practice opportunities, including outpatient examples, and appreciation of the benefits of observing interprofessional teamwork.

## Discussion

SAVE training is a novel curriculum that allows interprofessional ad hoc teams to perform medical management and de-escalation of a caregiver in a safe, realistic hospital setting. Typically, training focuses on only one of these aspects, but in practice, clinicians must be adept at managing both simultaneously, and often the worsening clinical status of the child leads caregivers to become distressed. This curriculum highlights how medical instability can escalate caregiver distress, and caregiver escalation can impair clinical care through communication breakdowns, safety concerns, or delayed interventions. Simulating this bidirectional relationship shows teams that addressing emotional and psychosocial needs is essential to medical care. Participants experienced how effective communication and empathy improve clinical outcomes while practicing attention triage, cognitive load management, and safe decision-making under stress. Further, participants recognized the importance of assigning roles, deferring to expertise, and teamwork in managing these challenging situations.

Facilitators encouraged hospital staff to apply the BEAR framework with SP feedback and reinforced verbal de-escalation resources, such as unit social workers, chaplains, charge nurses, nurse managers, and Code SWIFT team members. The ombudsman (patient advocate) and nurse navigators are resources that could be contacted later.

Participants demonstrated significant improvements in use of the Bond and Reinforce communication tools from the first to second scenarios. During debriefing, participants shared that introductions were often overlooked with upset caregivers, while Assessing and Engaging were more commonly used and felt easier. SPs noted after the first session that introductions and clarifying at bedside the individual's relationship to the patient may have led to improvements in the second scenario. Further, debriefing reinforced the importance of Bonding with the caregiver to establish trust necessary to de-escalate the situation and Reinforcing next steps.

Although group simulations may have reduced individual involvement in verbal de-escalation, we observed that teams frequently collaborated to address the SP's concerns, with participants fluidly balancing patient care tasks and caregiver communication as the scenarios unfolded. For example, the bedside nurse may start by asking the parent's name and concerns while providing patient care, with another nurse, social worker, or nonclinical team member providing family support. Staff trained in mindfulness engaged the SP in breathing exercises, which was a useful approach. Nonclinical staff or direct observers for behavioral health patients sometimes felt helpless to assist the deteriorating patient but were able to use simple language to address and effectively calm the caregiver (the SP). Despite varying education and experience, participants’ diversity improved team management of de-escalation, and debriefings offered valuable perspectives on effective and ineffective clinical approaches.

Lessons learned included empowering all staff to engage with caregivers, showing care through simple gestures such as offering a chair, snack, or drink to support caregivers’ needs. Calling security was a question that arose in many debriefings, with staff opting to try other approaches first, although we encouraged staff to call security for any concerns about physical safety. While many clinicians reported experiencing prior episodes of verbal escalation, few had submitted safety event reports. We encouraged reporting to improve awareness and help secure additional resources.

As an academic center with a robust simulation team and access to a university SP program, we were fully equipped to deliver this training. In centers without these resources, simulations can be done in classrooms with low-fidelity manikins, as the focus is on communication. Staff members could be trained to respond if SPs are not available. We examined staff biases by asking how they might respond to caregivers with varying language, gender, or physical characteristics not displayed by the SPs.

This simulation did not address the complexities of situations requiring an interpreter. Our scenarios focused on the acute care setting and caregiver frustration over the decompensation of their child. Participants noted families frequently experience unresolved frustrations, such as long emergency department and inpatient bed wait times. De-escalating caregivers in outpatient settings is challenging due to limited resources; we aim to address this group in next steps. Here we have shared strategies to overcome generalizability to health centers without access to SPs or high-fidelity manikins. There were limitations in our QR code–based evaluation approach, including Wi-Fi challenges, not bringing cell phones to class, and incomplete responses. A limitation with participant and facilitator survey completion is sampling or response bias, including social desirability. Furthermore, a limitation of this curriculum is the lack of long-term follow-up on application of the BEAR framework in patient care.

SAVE training provided clinicians a meaningful opportunity to practice managing patient deterioration and using de-escalation skills, while assigning roles for both patient and caregiver support. There is a paucity of literature on de-escalation training for teams within pediatric facilities, with education focused on disciplines rather than health care teams. We designed the curriculum to equip staff with practical tools to address increasing incidents of workplace verbal violence. One future direction is adapting this scenario for ambulatory settings in a virtual training that would address parental frustration over wait times with the use of a chatbot to practice these scenarios. The results of SAVE training suggest that training on an organizational level may be effective in enhancing individual and team communication with caregivers, minimizing incidents of verbal violence and improving patient care.

## Appendices


Simulation Cases.docxSP Case.docxLearner Guide.pdfFacilitator Guide.docxTraining Slides.pptxTechnical Support Checklist.docxFlyer.pdfFeedback Survey.pdfFacilitator Debrief Worksheet.pdfPresurvey.pdf
*All appendices are peer reviewed as integral parts of the Original Publication.*
