# Supplementary material for: Simulation Addressing Verbal Escalation (SAVE): An Interprofessional Simulation for Pediatric Health Care Professionals
Source: MedEdPORTAL. 2026 Apr 15;22:11593. doi: 10.15766/mep_2374-8265.11593 (PMC13080524; doi:10.15766/mep_2374-8265.11593)
Supplement: Supplementary file 1 — Simulation Cases.docxSP Case.docxLearner Guide.pdfFacilitator Guide.docxTraining Slides.pptxTechnical Support Checklist.docxFlyer.pdfFeedback Survey.pdfFacilitator Debrief Worksheet.pdfPresurvey.pdf [file mep_2374-8265.11593-s001.zip › G. Flyer.pdf]

Simulation Addressing Verbal Escalation (SAVE) Training

Children's National.

Children's National Simulation Program

Multiple sessions starting October 2024

Target Audience: CNH nurses, physicians, advanced practice providers, technicians, unit support associates (USAs), social workers, respiratory therapists, UCAs, child life specialists, pharmacists, and others with clinical-facing roles

Narrative/Learning Objectives:

- Apply evidence-based management of sepsis
- Apply communication techniques to address verbal escalation of parent/caregiver
- Utilize appropriate resources available at Children's National for behavioral escalation events

Joint Accreditation: In support of improving patient care, this activity has been planned and implemented by Children's National Hospital. Children's National Hospital is jointly accredited by the Accreditation Council for Continuing Medical Education (ACCME), the Accreditation Council for Pharmacy Education (ACPE), and the American Nurses Credentialing Center (ANCC), to provide continuing education for the healthcare team. Children's National Hospital Accreditation Provider# 4008362

Credit Designation:

Children's National Hospital designates this live activity for a maximum of 1.0 ***AMA PRA Category 1 Credits™*** for physicians. Physicians should claim only the credit commensurate with the extent of their participation in the activity.

Children's National Hospital designates this activity for a maximum of 1 LIVE/Enduring **ANCC** contact hours.

Children's National Health System is accredited by the Accreditation Council for Pharmacy Education (ACPE) as a provider of **continuing pharmacy education.** This program meets ACPE criteria for 1 contact hour (0.1 CEU). Credit will be awarded through the CPE Monitor, within 3–4 weeks of the seminar to those who successfully complete the program and complete the online evaluation (to be sent within a week of CE activity completion). NABP number and month/day of birth are required to receive credit. This activity will expire within 30 days of activity.

As a Jointly Accredited Organization, Children's National Hospital is approved to offer social work continuing education by the Association of Social Work Boards (ASWB) Approved Continuing Education (ACE) program. Organizations, not individual courses, are approved under this program. Regulatory boards are the final authority on courses accepted for continuing education credit. Social workers completing this course receive **1 general continuing education credits**.

JOINTLY ACCREDITED PROVIDER™
INTERPROFESSIONAL CONTINUING EDUCATION

ce.childrensnational.org

Scan QR code to sign up or click here

For questions, email simulation@childrensnational.org
