# Supplementary material for: Simulation Addressing Verbal Escalation (SAVE): An Interprofessional Simulation for Pediatric Health Care Professionals
Source: MedEdPORTAL. 2026 Apr 15;22:11593. doi: 10.15766/mep_2374-8265.11593 (PMC13080524; doi:10.15766/mep_2374-8265.11593)
Supplement: Supplementary file 1 — Simulation Cases.docxSP Case.docxLearner Guide.pdfFacilitator Guide.docxTraining Slides.pptxTechnical Support Checklist.docxFlyer.pdfFeedback Survey.pdfFacilitator Debrief Worksheet.pdfPresurvey.pdf [file mep_2374-8265.11593-s001.zip › H. Feedback Survey.pdf]

Training Feedback Survey

Please complete the survey below before claiming Continuing Education Credit.

Upon completion you will be directed to the OPTIONAL SAVE Training Evaluation Post-Survey (Research). You may close out of the second survey if you do not wish to participate.

Thank you!

	Nothing/Not at all	A little bit	Some	Quite a bit
How much did you learn in today's educational activity?	◯	◯	◯	◯
How engaged were you in this educational activity?	◯	◯	◯	◯
How effective were the facilitators?	◯	◯	◯	◯

Did you detect any conflict of interest, bias, commercial support, or product endorsement?

◯ Yes
◯ No

Please explain:

____

After attending the educational activity, please share what you learned that was meaningful to your practice.

____

What worked well about this educational activity?

____

Please share any comments about how we can improve this educational activity.

____
