# Supplementary material for: Simulation Addressing Verbal Escalation (SAVE): An Interprofessional Simulation for Pediatric Health Care Professionals
Source: MedEdPORTAL. 2026 Apr 15;22:11593. doi: 10.15766/mep_2374-8265.11593 (PMC13080524; doi:10.15766/mep_2374-8265.11593)
Supplement: Supplementary file 1 — Simulation Cases.docxSP Case.docxLearner Guide.pdfFacilitator Guide.docxTraining Slides.pptxTechnical Support Checklist.docxFlyer.pdfFeedback Survey.pdfFacilitator Debrief Worksheet.pdfPresurvey.pdf [file mep_2374-8265.11593-s001.zip › I. Facilitator Debrief Worksheet.pdf]

SAVE Training Facilitator Worksheet

**Date:**	**Session Information**
**# Learners**	
**Which SP?**	A B C D E F G
**Interprofessional?**	YES or NO
If no, why not?	
**Technical Difficulties?**	
**What went well/could be improved/comments for leadership/ interesting topics in debriefs?**	
**SCENARIO 1**	
**Which components of BEAR were used?**	Steps used:<br>[ ] Bond – Introductions<br>[ ] Engage – Validate feelings/set expectations<br>[ ] Assess – Check-ins/manage expectations<br>[ ] Reinforce – Summarize
**Did team mobilize:**	[ ] Clinical Escalation (Rapid Response Team, Staff Assist, Code Blue, etc.)<br>[ ] SW<br>[ ] SWIFT (SW Intervention for Families and Teams)<br>[ ] Security<br>[ ] Other: _________
**SCENARIO 2**	
**Which components of BEAR were used?**	Steps used:<br>[ ] Bond – Introductions<br>[ ] Engage – Validate feelings/set expectations<br>[ ] Assess – Check-ins/manage expectations<br>[ ] Reinforce – Summarize
**Did team mobilize:**	[ ] Clinical Escalation (Rapid Response Team, Staff Assist, Code Blue, etc.)<br>[ ] SW<br>[ ] SWIFT (SW Intervention for Families and Teams)<br>[ ] Security<br>[ ] Other: _________
