# Supplementary material for: Simulation Addressing Verbal Escalation (SAVE): An Interprofessional Simulation for Pediatric Health Care Professionals
Source: MedEdPORTAL. 2026 Apr 15;22:11593. doi: 10.15766/mep_2374-8265.11593 (PMC13080524; doi:10.15766/mep_2374-8265.11593)
Supplement: Supplementary file 1 — Simulation Cases.docxSP Case.docxLearner Guide.pdfFacilitator Guide.docxTraining Slides.pptxTechnical Support Checklist.docxFlyer.pdfFeedback Survey.pdfFacilitator Debrief Worksheet.pdfPresurvey.pdf [file mep_2374-8265.11593-s001.zip › J. Presurvey.pdf]

Pre-Survey

Survey Information:

This survey is optional research to understand the impact of the SAVE training. Participating in the surveys is optional and not required to complete the training. We will use your email to link your survey responses over time, but will deidentify your surveys by removing your email address before analyzing the data. Prior to completing the survey, please review the Study Information Sheet at the beginning of the survey. Continuing past the information sheet signifies your consent to participate in this study.

Survey Instructions:

Please take 5 minutes to respond to this survey.
Please do not identify yourself or others in open-ended responses.

Study Information Sheet:

[Attachment: "SAVE Study Information Sheet for Participants V2.pdf"]

Section 1:

Please enter your email (use the same address for all SAVE surveys) : ________________________________

What is your age?

- ◯ < 20
- ◯ 20-29
- ◯ 30-39
- ◯ 40-49
- ◯ 50-59
- ◯ 60-69
- ◯ 70-79
- ◯ 80-89
- ◯ 90+

Which best describes your Gender? - We recognize there are multiple gender identities but have listed these 5 options for simplicity with a free-response option.

- ◯ Female
- ◯ Male
- ◯ Non-binary
- ◯ I prefer not to answer
- ◯ Free response: ____

Gender: ________________________________

Race/Ethnic Identity (check all that apply):

- ☐ American Indian or Alaska Native
- ☐ Asian
- ☐ Black or African American
- ☐ Middle Eastern/North African
- ☐ White
- ☐ Hispanic/Latino
- ☐ Decline to Identify

Which of the following best describes your role?

- ◯ RN
- ◯ Physician (MD, DO, etc.)
- ◯ Advanced Practice Provider (NP, PA, AA, etc.)
- ◯ Direct Observer (USA, PCT, CPS, Technicians, etc.)
- ◯ Not Listed Above (RT, SW, Pharmacy, UCA, Child Life, Student, etc.)

Please Specify:	______________________
How long (in years) have you been in your current profession? If < 6 months, enter "0".	______________________
How long (in years) have you been working at Children's National Hospital? (If < 6 months, enter "0").	______________________
Which of the following best describes your primary clinical work setting?	◯ Acute Care Inpatient<br>◯ Ambulatory (Primary and Specialty)<br>◯ Critical Care (CICU, PICU, NICU)<br>◯ Emergency Department<br>◯ Perioperative Services (Pre-Op, OR, PACU)<br>◯ Non-Clinical<br>◯ Not Listed Above
Please Specify:	______________________
Section 2:	

The following three items ask about your feelings of safety while working in the hospital.

	1 - Strongly Disagree	2	3	4	5 - Strongly Agree
I feel safe (free from violence) when working in the hospital.	◯	◯	◯	◯	◯
I think there is a good chance of being injured from an assault by a patient while working in the hospital in the next 6 months.	◯	◯	◯	◯	◯
I think there is a good chance of being injured by a visitor while working in the hospital during the next 6 months.	◯	◯	◯	◯	◯

The following four items ask you to describe how confident you are in handling visitors who become aggressive.

	1 - Not Confident	2	3	4	5 - Extremely Confident
Your ability to manage a visitor's agitation or verbal aggression?	◯	◯	◯	◯	◯
Your ability to manage visitors who become verbally abusive to you?	◯	◯	◯	◯	◯

Your ability to manage visitors who physically threaten you or your co-workers?	◯	◯	◯	◯	◯
Your ability to manage visitors who become physically violent towards you or your co-workers?	◯	◯	◯	◯	◯

Have you experienced verbal violence from a parent, caregiver, or visitor in the past 3 months?
◯ Yes
◯ No

How many times?
____

Did you utilize any of the following tools/teams available at Children's (select all that apply)?
☐ Call Charge RN/Physician/Manager
☐ Call Unit/Clinic Social Worker
☐ SWIFT
☐ Call Security
☐ Other

Other
____

If you did not utilize any of the above tools/teams, why not?
____

Did you file a Safety Event Report?
◯ Yes
◯ No

If you did not file a Safety Event Report, why not?
____

Do you think you could have prevented verbal escalation?
◯ Yes
◯ No

Why or why not?
____

How much do you agree with the following statements regarding your own clinical interactions?

	Strongly Disagree	Disagree	Agree	Strongly Agree
I consistently seek out the reasons why a parent/caregiver/visitor exhibited verbal escalation	◯	◯	◯	◯

I consistently reflect on my own responses/behaviors when encountering a verbal escalation situation involving a parent/caregiver/visitor	○	○	○	○
I tend to avoid situations in which I have experienced a previous verbal escalation	○	○	○	○

Section 3:

Please use this space for any comments or concerns: __________________________________
