# Supplementary material for: Simulation Addressing Verbal Escalation (SAVE): An Interprofessional Simulation for Pediatric Health Care Professionals
Source: MedEdPORTAL. 2026 Apr 15;22:11593. doi: 10.15766/mep_2374-8265.11593 (PMC13080524; doi:10.15766/mep_2374-8265.11593)
Supplement: Supplementary file 1 — Simulation Cases.docxSP Case.docxLearner Guide.pdfFacilitator Guide.docxTraining Slides.pptxTechnical Support Checklist.docxFlyer.pdfFeedback Survey.pdfFacilitator Debrief Worksheet.pdfPresurvey.pdf [file mep_2374-8265.11593-s001.zip › A. Simulation Cases.docx]

| **Appendix A: *MedEdPORTAL* Simulation Case Template**  **SIMULATION CASE TITLE: SAVE Training Scenario 1**  **AUTHORS: Simranjeet Sran MD MEd CHSE, Heather Walsh PhD RN PCNS-BC CHSE-A CPN**  **LEARNER AUDIENCE: Interprofessional hospital-based staff (physicians, nurses, social workers, therapists, technicians)** | |
| --- | --- |
| **PATIENT NAME: Simon Jones**  **PATIENT AGE: 11mo**  **CHIEF COMPLAINT: Decreased responsiveness**  **PHYSICAL SETTING: Acute Care Floor/ED** | |
|  | |
| **Brief Narrative Description of Case** | *Include the presenting patient chief complaint and overall learner goals for this case.*  In a case that highlights an infant with bronchiolitis concerning for sepsis, the learner works to apply evidence-based management of sepsis while addressing the concerns of a caregiver using communication techniques to address verbal escalation of parent/caregiver. Identification and utilization of appropriate resources locally available for behavioral de-escalation that can then be applied to real-life scenarios. |
| **Primary Learning Objectives** | *What should the learners gain in terms of knowledge and skill from this case? Use action verbs and utilize Bloom’s Taxonomy as a conceptual guide.*   1. Apply evidence-based management of sepsis 2. Apply communication techniques to address verbal escalation of parent/caregiver 3. Utilize appropriate resources available at Children’s National for behavioral escalation events |
| **Critical Actions** | *List which steps the participants should take to successfully manage the simulated patient. These should be listed as concrete actions that are distinct from the overall learning objectives of the case.*   1. Identify sepsis 2. Treat sepsis with antipyretics, a fluid bolus, antibiotics, and increasing oxygen support 3. Establish rapport with the parent using the BEAR framework 4. Use ancillary staff such as social work, chaplain or child life, to support the parent 5. Escalate clinical care (e.g., staff assist, rapid response) |
| **Learner Preparation or Prework** | *What information should the learners be given prior to initiation of the case?*  Weight: 8 kg  It is change of shift and you have just received a brief handoff: Simon Jones is an 11mo M with acute viral bronchiolitis who has been in the hospital for 24 hours and is currently on 2L nasal cannula. You are on the acute care floor (could be the ED depending on the learners). You are in the unit hallway as the scenario begins |

| Initial Presentation | | | |
| --- | --- | --- | --- |
| **Initial Vital Signs** | T 35 C rectal  HR 200, sinus  RR 70  BP 75/35 (48)  O2 Sat 95% | | |
| **Overall Setting and Appearance** | *What do learners encounter when they first enter the room? What environment are the learners in? What is the appearance of the mannequin?*  Learner is in the hallway as the case begins. Parent calls the nurse into the room. The patient (toddler manikin) is in a crib and is attached to the monitor, with the parent at bedside. PIV is in place. The monitor is beeping. | | |
| **Standardized Participants (and Their Roles in the Room at Case Start)** | *Who is present at the beginning and what is their role? Who may play them? Describe what they should say (i.e., their verbal scripts).*  The parent is at bedside and starts the case by calling a nurse into the room. The parent is concerned Simon is less interactive. Asks pertinent questions like “Is Simon going to be okay?” | | |
| **HPI** | SP provides background HPI and medical history. | | |
| **Past Medical/Surgical History** | **Medications** | **Allergies** | **Family History** |
|  | Multivitamin | None | None |
| **Physical Examination** | | | |
| **General** | Moaning, ill appearing | | |
| **HEENT** | Eyes closed | | |
| **Neck** | No lymphadenopathy | | |
| **Lungs** | Coarse lung sounds, subcostal retractions | | |
| **Cardiovascular** | Tachycardic, cap refill 4-5 seconds | | |
| **Abdomen** | Soft, nondistended | | |
| **Neurological** | Minimal movement | | |
| **Skin** | Mottled | | |
| **GU** | Normal GU exam | | |
| **Psychiatric** | N/A | | |

| Instructor Notes - Changes and CASE Branch Points  *This section should be a list with detailed description of each step that may happen during the case. If medications are given, what is the response? Do changes occur at certain time points? Should the nurse or other participant prompt the learners at given points? Should new actors or participants enter, and when? Are there specific things the patient will say or do at given times? There are a few examples given, but it is expected that most cases will have many more changes and potential branch points.*  *If you have a more complex branching algorithm than can be accommodated by the structure below, feel free to replace this section with your own. Review some recent simulation publications on MedEdPORTAL for examples.* | | |
| --- | --- | --- |
| **Intervention / Time Point** | **Change in Case** | **Additional Information** |
| Start of Case – SP calls participant into the room | Participants assess patient, identifies sepsis, calls provider | RN alerts the provider: “Doctor, the blood pressure is 75/35, HR 200”  Physical exam description can be provided when asked:   - moaning, minimal movement - cap refill 4-5 sec - mottling - subcostal retractions - abdomen soft, non-distended |
| 3 minutes | Provider identifies sepsis, asks for fluids, antibiotics, start oxygen and fever control | Can provide labs when asked: WBC 18 Hgb 10 HCT 30 Plt 300, neutrophils 70% Bands 5%, CXR normal |
|  | Parent asks for updates in the plan, asks questions like “Is Simon going to be ok?” |  |
| 5 minutes – Decompensation | Despite antipyretics and oxygen repeat vitals:  T 35  HR 190  RR 70  BP 60/25 (35)  O2 Sat 92% | Participants escalate care (if on acute care, calls for ICU)  If asked for physical exam: lethargic, responds to  painful stimulus  Still  tachypneic/tachycardic |
|  | As more people enter the room, caregiver gets more concerned, asking more questions and should express lack of understanding in plan | May assign a team member to communicate with the parent, parent will become more understanding with addition of chaplain, social work or child life |
| Participant prioritizes fluids with push pull | HR and BP downtrend |  |
| 5 minutes: Participant completes push pull bolus | Improved capillary refill and respiratory status | HR 150, RR 40, BP 80/60  Case is completed |

**Ideal Scenario Flow**

*Provide a detailed narrative description of the way this case should flow if participants perform in the ideal fashion.*

The learners enter the room when summoned by the parent to find a patient in respiratory distress with delayed cap refill. They immediately place the patient on bedside monitors and recognize that the patient is tachycardic and hypotensive, suggesting sepsis. Antipyretics and antibiotics are given. Supplemental oxygen is provided and an IV fluid bolus is ordered. The parent is initially worried but is able to provide the child’s medical history and events leading up to hospitalization. The patient’s respiratory distress improves but does not resolve, and hypotension is refractory to IV fluids. After completing a physical examination and obtaining an appropriate history, the providers note that the patient’s hypotension has continued to worsen and ultimately push pull bolus is required with an escalation of the care team. Throughout the case, the parent is concerned and asks for reassurance about her child’s care. As more people enter the room, the parent becomes more distressed and reiterates her need to know the plan and what is happening to her child. Chest x-ray is normal, laboratory studies (if obtained) demonstrate an elevated white count with neutrophilic predominance and bandemia. The patient’s tachycardia, respiratory distress and hypotension resolve as a push-pull bolus are administered. The parent calms as staff work to discuss the plan with her and address her concerns.

**Anticipated Management Mistakes**

*Provide a list of management errors or difficulties that are commonly encountered when using this simulation case.*

1. Failure to respond to the caregiver: As many simulations focus primarily on medical management, we found when using this case that some of our learners focused only on the medical aspects of the case and did not appropriately address the caregiver’s concerns or tried to separate the escalated caregiver from the patient. We found that after reviewing de-escalation techniques before the second case, participants were able to address both the medical and social aspects in the second case.
2. Failure to medically manage the patient: When teams were composed of newer staff or teams were composed without a nurse or physician role, some teams struggled with management of sepsis and the need for fluid resuscitation. To address this, one of the facilitators stepped in to portray the nurse or physician to assume those tasks, allowing the ad hoc team to focus on addressing the parent.

| **SIMULATION CASE TITLE: SAVE Training Scenario 2**  **AUTHORS: Simranjeet Sran MD MEd CHSE, Heather Walsh PhD RN PCNS-BC CHSE-A CPN**  **LEARNER AUDIENCE: Interprofessional hospital-based staff (physicians, nurses, social workers, therapists, technicians)** | |
| --- | --- |
| **PATIENT NAME: Simon Jones**  **PATIENT AGE: 2yo**  **CHIEF COMPLAINT: Monitor Alarming**  **PHYSICAL SETTING: Acute Care Floor/ED** | |
|  | |
| **Brief Narrative Description of Case** | *Include the presenting patient chief complaint and overall learner goals for this case.*  In a case that highlights a patient with pneumonia with concern for sepsis, the learner works to apply evidence-based management of sepsis while addressing the concerns of a caregiver using communication techniques to address verbal escalation of parent/caregiver. Building on the skills solidified in the first case, learners use de-escalation tactics to address an angry parent. |
| **Primary Learning Objectives** | *What should the learners gain in terms of knowledge and skill from this case? Use action verbs and utilize Bloom’s Taxonomy as a conceptual guide.*   1. Recognize uncompensated septic shock 2. Apply management of sepsis to a decompensating patient 3. Use the BEAR framework with a frustrated parent 4. Reflect on attitudes and perceptions that made the case difficult |
| **Critical Actions** | *List which steps the participants should take to successfully manage the simulated patient. These should be listed as concrete actions that are distinct from the overall learning objectives of the case.*   1. Identify sepsis 2. Treat sepsis with antipyretics, a fluid bolus, antibiotics, and oxygen 3. Establish a relationship with the parent using the BEAR framework 4. Use ancillary staff such as social work, chaplain or child life, to support the parent 5. Escalate care to the ICU |
| **Learner Preparation or Prework** | *What information should the learners be given prior to initiation of the case?*  Age: 2 yo M  Weight: 12 kg  Simon has returned to the hospital now as a 2-year-old admitted with flu and pneumonia who is tachycardic, tachypneic, and febrile. Again, it is change of shift and you have just received a brief handoff. You are in the unit hallway as the scenario begins. |

| Initial Presentation | | | |
| --- | --- | --- | --- |
| **Initial Vital Signs** | T 39 C  HR 160  RR 50  BP 75/35 (48)  O2 Sat 95% on 2 L NC | | |
| **Overall Setting and Appearance** | *What do learners encounter when they first enter the room? What environment are the learners in? What is the appearance of the mannequin?*  Learner is in the hallway as the case begins. Parent calls the nurse into the room. The patient is in a bed, not attached to the monitor, with the parent at bedside. IV is in place and he is on NC. | | |
| **Standardized Participants (and Their Roles in the Room at Case Start)** | *Who is present at the beginning and what is their role? Who may play them? Describe what they should say (i.e., their verbal scripts).*  The parent is at bedside and starts the case by calling a nurse into the room. The parent is concerned Simon isn’t acting like himself. The caregiver is visibly annoyed/irritated. | | |
| **HPI** | SP provides background HPI and medical history. | | |
| **Past Medical/Surgical History** | **Medications** | **Allergies** | **Family History** |
|  | Multivitamin | None | None |
| **Physical Examination** | | | |
| **General** | Moaning, ill appearing | | |
| **HEENT** | Eyes closed | | |
| **Neck** | No lymphadenopathy | | |
| **Lungs** | Coarse lung sounds, subcostal retractions | | |
| **Cardiovascular** | Tachycardic, cap refill 4-5 seconds | | |
| **Abdomen** | Soft, nondistended | | |
| **Neurological** | Minimal movement | | |
| **Skin** | Mottled | | |
| **GU** | Normal GU exam | | |
| **Psychiatric** | N/A | | |

| Instructor Notes - Changes and CASE Branch Points  *This section should be a list with detailed description of each step that may happen during the case. If medications are given, what is the response? Do changes occur at certain time points? Should the nurse or other participant prompt the learners at given points? Should new actors or participants enter, and when? Are there specific things the patient will say or do at given times? There are a few examples given, but it is expected that most cases will have many more changes and potential branch points.*  *If you have a more complex branching algorithm than can be accommodated by the structure below, feel free to replace this section with your own. Review some recent simulation publications on MedEdPORTAL for examples.* | | |
| --- | --- | --- |
| **Intervention / Time Point** | **Change in Case** | **Additional Information** |
| Start of Case – SP calls participant into the room | Participant assess patient, identifies sepsis, calls provider | RN alerts the provider: “Doctor, the blood pressure is 75/35, HR 160”  Physical exam description can be provided when asked:   - moaning, minimal movement - cap refill 4-5 sec - mottling - subcostal retractions - abdomen soft, non-distended |
| 3 minutes | Provider identifies sepsis, asks for fluids, antibiotics, oxygen and antipyretic | Can provided labs when asked: WBC 18 Hgb 10 HCT 30 Plt 300, neutrophils 70% Bands 5%, CXR with right focal consolidation |
|  | Parent maneuvers to get to the patient, stating things like “you are hurting my child” |  |
| 5 minutes – Decompensation | Despite antipyretics and oxygen repeat vitals:  T 35  HR 190  RR 70  BP 60/25 (35)  O2 Sat 92% | Participants escalate care (if on acute care, calls for ICU)  If asked for physical exam: lethargic, responds to  painful stimulus  Still  tachypneic/tachycardic |
|  | As more people enter the room, caregiver exhibits verbal escalation, may threaten to take Simon AMA | Team should identify parent escalation, identify appropriate staff for family communication and identify resources to help the family |
| Participant prioritizes fluids with push pull | HR and BP downtrend |  |
| 5 minutes: Participant completes push pull bolus | Improved capillary refill and respiratory status | HR 150, RR 40, BP 80/60  Case is completed |

**Ideal Scenario Flow**

*Provide a detailed narrative description of the way this case should flow if participants perform in the ideal fashion.*

The learners enter the room when summoned by the parent to find a patient in respiratory distress with delayed cap refill. They immediately place the patient on bedside monitors and recognize that the patient is tachycardic and hypotensive. Antipyretics and antibiotics are given. Supplemental oxygen is continued and an IV fluid bolus is ordered. The parent is initially worried, but quickly escalates with concern for their child. The caregiver repeatedly tries to get closer to the patient, stating that the team is hurting him and this wouldn’t have happened if they hadn’t waited so long for a bed. The patient’s respiratory distress improves but does not resolve, and hypotension is refractory to IV fluids. After completing a physical examination and obtaining an appropriate history, the providers note that the patient’s hypotension has continued to worsen and ultimately push pull bolus is required with an escalation of the care team. As more people enter the room, the parent becomes more distressed and threatens to take the patient from the hospital AMA. Chest x-ray is with right sided opacification, laboratory studies (if obtained) demonstrate an elevated white count with neutrophilic predominance and bandemia. The patient’s tachycardia, respiratory distress and hypotension resolve as a push-pull bolus are administered. The parent calms as staff work to discuss the plan with her and address her concerns.

**Anticipated Management Mistakes**

*Provide a list of management errors or difficulties that are commonly encountered when using this simulation case.*

*For example:*

1. Failure to maintain emotions in check: As the caregiver was much more escalated in this case, some groups struggled to maintain calm and tried to separate the caregiver from the situation which made the caregiver escalate further. Addressing emotions and tactics for keeping situations calm or identifying team members who could specifically help support the child were specific points of the debrief.
2. Personal bias impacting caregiver interactions: As these situations were derived from real scenarios, some participants had prior experiences with how to respond, both positively and negatively. Addressing personal bias and posing questions for how the situation would change based on different demographic factors of caregivers was a focal point of the debrief.
