# Supplementary material for: Simulation Addressing Verbal Escalation (SAVE): An Interprofessional Simulation for Pediatric Health Care Professionals
Source: MedEdPORTAL. 2026 Apr 15;22:11593. doi: 10.15766/mep_2374-8265.11593 (PMC13080524; doi:10.15766/mep_2374-8265.11593)
Supplement: Supplementary file 1 — Simulation Cases.docxSP Case.docxLearner Guide.pdfFacilitator Guide.docxTraining Slides.pptxTechnical Support Checklist.docxFlyer.pdfFeedback Survey.pdfFacilitator Debrief Worksheet.pdfPresurvey.pdf [file mep_2374-8265.11593-s001.zip › B. SP Case.docx]

**Appendix B: Standardized Patient Case**

Date: 6/15/2024

Primary Case Author: Simranjeet S. Sran MD MEd CHSE

Secondary Case Author: Heather Walsh PhD RN PCNS-BC CHSE-A CPN

Standardized Patient Educator: Jennifer Owens MAEd

Name of Case: SAVE Training Scenario 1

Name of Educational and/or Assessment Activity: SAVE Training

Patient Name: Simon Jones /parent Peyton Jones

Chief Complaint: Infant admitted with viral bronchiolitis, now less responsive

Most Likely Diagnosis and Differential with Rationale from History and/or Physical Exam:

Sepsis secondary to bronchiolitis - Simon Jones is a 11-month-old M with acute viral bronchiolitis who was admitted 24 hours ago on 2L nasal cannula. He had been doing well until recently when the caregiver called out of the room for assistance and concerned because he is less responsive.  An ad hoc team of clinicians from the hallway respond at change of shift and upon assessment, note he is hypothermic, hypotensive, and tachycardic, indicating septic shock.

Challenge Question:

Mother to keep trying to get information if it is not shared with you. As staff enter the room, if introductions are not done, ask who they are and what they are doing. As more people enter the room, SP to demonstrate more concern. If ignored, ask what is happening and speak more loudly to get attention. If medical jargon is being used, ask for clarification (e.g., What does tachycardia mean? What is a rapid response/ the ICU?) If team discusses medication and treatment without explanation, ask questions (e.g., What is sepsis? Is Simon going to be okay?). If the responders provide information in simple terms, SP can continue to ask questions as appropriate.

Domains: Check all that apply

- Professionalism X
- Communication and Interpersonal Skills X
- Medical History
- Physical Exam X
- Shared Decision-Making
- Patient Education X
- Clinical Reasoning X
- Documentation
- Handoff
- Presentation
- Other:

Type and Level of Learner: Target audience was interprofessional clinicians, ranging from novice to expert, including nurses, physicians, advanced practice providers (APPs), respiratory therapists, social workers, child life specialists, speech and language pathologists, patient care technicians, unit clerical associates (clerks), and direct observers.

Case Objectives: Please list specific objectives for each of the domains you have checked above:

1. Apply evidence-based management of sepsis

2. Apply communication techniques to address verbal escalation of parent/caregiver

3. Utilize appropriate resources available locally for behavioral escalation events

**Scenario 1**

| SETTING: outpatient, in patient, ED, home, nursing home, rehab, group, etc. | Acute Care Floor or ICU/ED |
| --- | --- |
| PATIENT PROFILE: Information about the “patient” that helps select an SP and helps the learner get an understanding of them as a person. SP will know more information about the patient than learner will ever ask but allows SP to portray a fully developed patient personality. If none of the items below are particulars for the case, please write “all may be used.” | |
| Age range | 6 wo/25-45 years |
| Religious/spiritual background | Spiritual |
| Sex (e.g., male, female, intersex, transwoman, transman) | Male/Female |
| Sexual orientation (e.g., heterosexual, lesbian, gay, bisexual, pansexual, queer, asexual) | All may be used |
| Gender expression (e.g., man, woman, genderqueer) | All may be used |
| Race and ethnicity (e.g., to promote educational diversity, we use a diverse pool of SPs.) | All may be used |
| Physical description (e.g., BMI, height range) | All may be used |
| Physical limitations | All may be used |
| Patient appearance (e.g., disheveled, hospital gown, business casual, casual) | Worried, first-time parent; had never been in a hospital before except to give birth to their now 11-month-old |
| Moulage + location (e.g., none, bruises, scars, body piercing, tattoos) | N/A |
| Affect (e.g., pleasant, cooperative) | Concerned at son’s current condition (less responsive) |
| Family group (e.g., who is family, who they live with) | Lives with both parents |
| Education | Not specified |
| Level of health literacy | College (can be adapted) |
| Employment, if any - present and past, noting any current stresses | Parents work for the federal government |
| Home/homeless - type of dwelling, number of stories, owned or rented | Have a home- not specified whether apartment or house, own or rent |
| Financial situation - any current stresses | N/A |
| Insurance status (e.g., un/under/insured, public/private, HMO/PPO) | Private insurance |
| Habits (i.e., diet, exercise, caffeine, smoking, alcohol, drugs) | All may be used |
| Activities (i.e., hobbies, sports, clubs, friends) | All may be used |
| Typical day - what is the usual daily routine | Simon attends day care while his parents are at work; has experienced work of breathing and fever with decreased po intake and fewer wet diapers prior to hospitalization. |

| CASE INFORMATION | |
| --- | --- |
| Chief Concern: What the patient will say when greeted by the student. The patient’s primary reason for seeking medical care often stated in their own words. | “Can I get some help in here? I’m really concerned about my son. He feels cold and he looks worse than when we got here. He’s not moving very much. No one has explained to me what’s happening.” |
| Additional Concerns: Other, if any, concerns the patient has today (i.e., symptoms, requests, expectations, etc.) that will become part of set agenda. | N/A |
| THE PATIENT’S STORY: The SP will be asked to tell their symptom story and the personal and emotion impact for each of their concerns. You will want to write this in the patient’s voice. The symptom story should be able to answer this question: “Tell me more about [chief concern/additional concern], starting at the beginning and bringing me up to now.”    The personal context should be able to answer questions concerning the broader personal/psychosocial context of symptoms, especially the patient’s beliefs/attributions.    The emotional context should be able to ask how are you doing with this, how does this make you feel, how has this affected you emotionally? IMPACT: How has this affected your life? How has this been for your family? | Parent went to pediatrician yesterday because the patient wasn’t eating well and hadn’t had wet diapers with upper respiratory infection (URI) symptoms, and had some increased work of breathing.    Pediatrician sent parent to ED where he had lab tests done and was admitted for oxygen therapy.  His breathing improved with oxygen but now he looks worse.  Parent has been told he is admitted for “something that sounds like bronchitis, but it isn’t bronchitis” but has no idea what that is.    “He’s breathing fast and his chest is caving in. He looks worse than when we came in and isn’t moving.” |
| HISTORY OF PRESENT ILLNESS: Although some of the HPI will be given in the patient’s symptom story, the learners will expand the story during the direct question section. Below, describe the detailed history, usually about the chief concern, which the student must develop in order to make a useful assessment of the problem: | |
| Onset (when; gradual or sudden) | Symptoms started yesterday but acutely worsened suddenly |
| Setting (what was going on or where was patient when symptoms first noticed?) | Symptoms first started at home yesterday, but now is lying in the bed working much harder to breathe |
| Duration (how long) | A few minutes for the acute symptoms |
| Time relationships (frequency, constant or intermittent) | New onset, constant until interventions performed to treat sepsis |
| Location | N/A |
| Radiation | N/A |
| Quality | N/A |
| Amount | N/A |
| Aggravated by what | N/A |
| Relieved by what | N/A |
| Associated with what | N/A |
| Attitude (what does the patient think is the problem, and how do they feel about it) | Parent is very scared and worried |
| Overall course | He is worsening |
| REVIEW OF SYSTEMS: Significant positives and negatives | |
| + Cough | + Congestion |
| + Fever | + Work of breathing |
| No vomiting, diarrhea | + Poor po intake |
|  |  |
| Past medical history | Simon is a healthy full-term infant.  No surgeries. |
| Medication allergies (name and reaction) | None |
| Environmental allergies (name and reaction) | None |
| Illnesses | None |
| Vaccinations | Up to date |
| Surgeries | None |
| Accidents/injuries/trauma | None |
| Hospitalization | None |
|  | |
| Inclusive sexual and reproductive history | |
| Sexual practices  Sexual partners  Protection: Use of safer sex practices  Use of birth control if appropriate  Risk of intimate partner violence | All may be used |
| OB/GYN history | All may be used |
| Medications | Prescription/dose/reason: none  Over the counter/dose/reason: none  Herbs/supplements/dose/reason: Vit D drop daily, he is breastfed  Other: none |
| Immunizations |  |
| Tobacco products:   - Cigarettes - Cigar - Pipe - Chew - E-cigarettes | - Never X - Past - year started/year quit - Current - Quantity - # of years |
| Alcohol   - Beer - Wine - Liquor - Other | - Never X - Past - year started/year quit - Current - Quantity - # of years |
| Drugs   - Weed - Cocaine - Heroin - Meth - IV - Inhalants - Other | - Never X - Past - year started/year quit - Current - Quantity - # of years |
| Diet (describe) | Can be breast fed or take formula; poor oral intake before admission with few wet diapers noted |
| Exercise (describe) | None |
| List any other important social history or information important to this case | Parents have been married for 5 years. Safe at home, stable home situation; both parents work for the federal government. |
| Family history |  |
| Mother, father, siblings, grandparents, and other significant findings | None |
|  |  |
| Physical Exam - List exam maneuvers expected for this case and any abnormal findings that SP will simulate. (tenderness, hyper-hypo reflex, rebound, weakness, etc.) | |
| PHYSICAL EXAM FINDINGS |  |
| 1. Written in layperson’s terms |  |
| 1. General appearance - affect, appearance, position of patient at opening (i.e., sitting, lying down, holding abdomen, etc.) | Moaning, minimal movement |
| 1. Vital signs | T 35 C rectal  HR 200, sinus  RR 70  BP 75/35 (48)  O2 Sat 95% |
| 1. Specific findings and affect | cap refill 4-5 sec,  mottling  Subcostal retractions  Abdominal exam: soft,  non-distended |
| 1. Response to certain physical movements | As case progresses: Lethargic, responds to  painful stimulus  Still  tachypneic/tachycardic |
|  |  |
| DIAGNOSIS AND DIFFERENTIAL |  |
| Diagnosis with support from positive and negative history and PE findings | Concern for sepsis in the setting of viral bronchiolitis |
| Differential with support from positive and negative history and PE findings | Pneumonia, sepsis, worsening bronchiolitis |
|  |  |
| MANAGEMENT OR DIAGNOSTIC PLAN | ABC/ Pediatric assessment triangle  Actions:  • Assesses patient  • Identifies sepsis  • Asks for fluids  • Ask for antibiotics  • Asks for fever control (acetaminophen, ibuprofen)  • Start oxygen to help perfusion  • Prioritizes fluids with push pull  • If on acute care, calls for ICU (rapid response)  • Identifies escalating parent and asks for social worker, child life, or the chaplain  • If not already done, may assign a team member to help gather information  • May assign a team member to communicate with caregiver |
|  |  |
| PROFESSIONALISM ISSUES OR CHALLENGES |  |
|  | Family is overall concerned. “I don’t know what’s going on. I came here for help and he’s getting worse.”    *Scenario endpoint*   - If team explains the plan, the scenario will end   If no one explains the plan, parent to ask, “Can you tell me what’s going on?” |

**Scenario 2**

| SETTING: outpatient, in patient, ED, home, nursing home, rehab, group, etc. | Acute Care Floor or ICU/ED |
| --- | --- |
| PATIENT PROFILE: Information about the “patient” that helps select an SP and helps the learner get an understanding of them as a person. SP will know more information about the patient than learner will ever ask but allows SP to portray a fully developed patient personality. If none of the items below are particulars for the case, please write “all may be used.” | |
| Age range | Simon Jones 2yo/ Peyton Jones 25-45yo |
| Religious/spiritual background | Spiritual |
| Sex (e.g., male, female, intersex, transwoman, transman) | All may be used |
| Sexual orientation (e.g., heterosexual, lesbian, gay, bisexual, pansexual, queer, asexual) | All may be used |
| Gender expression (e.g., man, woman, genderqueer) | All may be used |
| Race and ethnicity (e.g., to promote educational diversity, we use a diverse pool of SPs.) | All may be used |
| Physical description (e.g., BMI, height range) | All may be used |
| Physical limitations | All may be used |
| Patient appearance (e.g., disheveled, hospital gown, business casual, casual) | All may be used |
| Moulage + location (e.g., none, bruises, scars, body piercing, tattoos) | All may be used |
| Affect (e.g., pleasant, cooperative) | Initially worried, becomes more angry as more people enter the room, threatens to leave as the case moves on |
| Family group (e.g., who is family, who they live with) | Safe at home, stable home situation; 6-month-old at home; parents stressed with 1 child at home and 1 in the hospital |
| Education | All may be used |
| Level of health literacy | All may be used |
| Employment, if any - present and past, noting any current stresses | Both parents work for the federal government |
| Home/homeless - type of dwelling, number of stories, owned or rented | All may be used |
| Financial situation - any current stresses | All may be used |
| Insurance status (e.g., un/under/insured, public/private, HMO/PPO) | All may be used |
| Habits (i.e., diet, exercise, caffeine, smoking, alcohol, drugs) | All may be used |
| Activities (i.e., hobbies, sports, clubs, friends) | All may be used |
| Typical day - what is the usual daily routine | All may be used |

| CASE INFORMATION | |
| --- | --- |
| Chief Concern: What the patient will say when greeted by the student. The patient’s primary reason for seeking medical care often stated in their own words. | Scenario begins with caregiver coming out of the room calling for help. |
| Additional Concerns: Other, if any, concerns the patient has today (i.e., symptoms, requests, expectations, etc.) that will become part of set agenda. | Caregiver is on edge because the patient has been in the ED for 19 hours waiting on a floor bed and was just admitted to acute care unit. Caregiver and patient have both not slept in 2 days. |
| THE PATIENT’S STORY: The SP will be asked to tell their symptom story and the personal and emotion impact for each of their concerns. You will want to write this in the patient’s voice. The symptom story should be able to answer this question: “Tell me more about [chief concern/additional concern], starting at the beginning and bringing me up to now.”    The personal context should be able to answer questions concerning the broader personal/psychosocial context of symptoms, especially the patient’s beliefs/attributions.    The emotional context should be able to ask how are you doing with this, how does this make you feel, how has this affected you emotionally? IMPACT: How has this affected your life? How has this been for your family? | Simon has a history of recurrent upper respiratory infections. Parent took the child to their pediatrician two days ago for fever and was diagnosed with flu. They were sent home with Oseltamivir and recommendations for supportive care (acetaminophen, ibuprofen, and maintaining hydration).    Parent then came to the ED when he was not improving and has been waiting for a floor bed for 19 hours. CXR revealed pneumonia and the patient required some oxygen.  The child has been mostly healthy although he was admitted for bronchiolitis at 11 months old.    Parent feels as though the child is getting worse and no one has checked on him.  As child decompensates, ask, “We’ve been in the ED for a day. This wouldn’t have happened if we were had gotten a room sooner. You’re starving my baby. He isn’t getting any fluid.  This is supposed to be a top hospital. This is bull shit.”    As staff use medical jargon, ask clarifying questions.  If placing an IV, ask “Why are you hurting my baby?”  “Who do I have to talk to get stuff done?”  “Is he in pain?” |
| HISTORY OF PRESENT ILLNESS: Although some of the HPI will be given in the patient’s symptom story, the learners will expand the story during the direct question section. Below, describe the detailed history, usually about the chief concern, which the student must develop in order to make a useful assessment of the problem: | |
| Onset (when; gradual or sudden) | Symptoms started 2 days ago |
| Setting (what was going on or where was patient when symptoms first noticed?) | Went to the pediatrician 2 days ago where he was diagnosed with the flu |
| Duration (how long) | 2 days |
| Time relationships (frequency, constant or intermittent) | Symptoms have worsened over the last two days. Parents brought him to the ED when he was not improving |
| Location | Not applicable |
| Radiation | Not applicable |
| Quality | Not applicable |
| Amount | Not applicable |
| Aggravated by what | Unsure |
| Relieved by what | Unsure |
| Associated with what | Unsure |
| Attitude (what does the patient think is the problem, and how do they feel about it) | Parents are worried he is worsening |
| Overall course | He has been worsening. |
| REVIEW OF SYSTEMS: Significant positives and negatives | |
| lethargic today/not active | No vomiting or diarrhea |
| not eating or drinking |  |
|  |  |
|  |  |
|  |  |
| Past medical history | Eczema  Hospitalized before for bronchiolitis but never in an ICU.  No previous surgeries. |
| Medication allergies (name and reaction) | None |
| Environmental allergies (name and reaction) | None |
| Illnesses | Bronchiolitis at 11 months of age, no other illnesses |
| Vaccinations | UTD |
| Surgeries | None |
| Accidents/injuries/trauma | None |
| Hospitalization | Hospitalized at 11 mo for bronchiolitis |
|  | |
| Inclusive sexual and reproductive history | |
| Sexual practices  Sexual partners  Protection: Use of safer sex practices  Use of birth control if appropriate  Risk of intimate partner violence | Not applicable |
| OB/GYN history | Not applicable |
| Medications | Prescription/dose/reason: hydrocortisone cream, seasonal allergy medicine  Over the counter/dose/reason: none  Herbs/supplements/dose/reason: none  Other: none |
| Immunizations | UTD on vaccines |
| Tobacco products:   - Cigarettes - Cigar - Pipe - Chew - E-cigarettes | - Never X - Past - year started/year quit - Current - Quantity - # of years |
| Alcohol   - Beer - Wine - Liquor - Other | - Never X - Past - year started/year quit - Current - Quantity - # of years |
| Drugs   - Weed - Cocaine - Heroin - Meth - IV - Inhalants - Other | - Never X - Past - year started/year quit - Current - Quantity - # of years |
| Diet (describe) | Regular diet |
| Exercise (describe) | Not applicable |
| List any other important social history or information important to this case | None |
| Family history |  |
| Mother, father, siblings, grandparents, and other significant findings | None |
|  |  |
| Physical Exam - List exam maneuvers expected for this case and any abnormal findings that SP will simulate. (tenderness, hyper-hypo reflex, rebound, weakness, etc.) | |
| PHYSICAL EXAM FINDINGS |  |
| 1. Written in layperson’s terms |  |
| 1. General appearance - affect, appearance, position of patient at opening (i.e., sitting, lying down, holding abdomen, etc.) | Moaning, minimal movement |
| 1. Vital signs | T 39 C  HR 160  RR 50  BP 75/35 (48)  O2 Sat 95% on 2 L NC |
| 1. Specific findings and affect | Prolonged cap refill  Subcostal retractions |
| 1. Response to certain physical movements | As the case progresses, lethargic, responds to  painful stimulus  Still tachypneic/tachycardic  Delayed cap refill |
|  |  |
| DIAGNOSIS AND DIFFERENTIAL |  |
| Diagnosis with support from positive and negative history and PE findings | Uncompensated septic shock in the setting of pneumonia |
| Differential with support from positive and negative history and PE findings | Worsening pneumonia, bacteremia, respiratory failure |
|  |  |
| MANAGEMENT OR DIAGNOSTIC PLAN | ABC/ Pediatric assessment triangle.  Actions:  • Assesses patient  • Identifies sepsis  • Asks for fluids  • Ask for antibiotics  • Asks for fever control (acetaminophen, ibuprofen)  • Start oxygen to help perfusion  • Prioritizes fluids with push pull  • If on acute care, calls for ICU (rapid response)  • Identifies escalating parent and asks for social worker, child life, or the chaplain  • If not already done, may assign a team member to help gather information  • May assign a team member to communicate with Caregiver |
|  |  |
| PROFESSIONALISM ISSUES OR CHALLENGES | Family is significantly upset and agitated. Accuse people of hurting your child (e.g. “You’re hurting my child”, “What are you doing to him?  You are making him worse.” Threaten legal action if they hurt your child “If anything happens, I’m going to sue this place.” Threaten to leave against medical advice (AMA): “I’m leaving. This place is bogus. I’m taking my child and going someplace else.”    If the team does any of these things, parent to de-escalate   - Introduces themselves - Validates feelings - Manage expectations - Explains things in simple terms - Assigns someone to talk to you - Sits down with you - Uses calming voice   **Failure to do these interventions (above) results in continued verbal escalation/ agitation**    *Scenario endpoint: (after several minutes of verbal escalation)*   - If team addresses your concerns, will stop and debrief - If team does not address your concerns and you continue to verbally escalate, will stop and debrief |
