# Supplementary material for: Simulation Addressing Verbal Escalation (SAVE): An Interprofessional Simulation for Pediatric Health Care Professionals
Source: MedEdPORTAL. 2026 Apr 15;22:11593. doi: 10.15766/mep_2374-8265.11593 (PMC13080524; doi:10.15766/mep_2374-8265.11593)
Supplement: Supplementary file 1 — Simulation Cases.docxSP Case.docxLearner Guide.pdfFacilitator Guide.docxTraining Slides.pptxTechnical Support Checklist.docxFlyer.pdfFeedback Survey.pdfFacilitator Debrief Worksheet.pdfPresurvey.pdf [file mep_2374-8265.11593-s001.zip › C. Learner Guide.pdf]

LEARNER GUIDE FOR SAVE TRAINING

Today's scenarios focused on medical management of a decompensating child as well as management of an escalating parent. Below are some things to keep in mind for building trust and rapport with parents/caregivers and caring for a patient with suspected sepsis.

MANAGEMENT OF SEPSIS

RECOGNIZE AND VERBALIZE SEPSIS

- Tachycardia + signs of infection
- Poor perfusion
- Mental status changes
- Hyperthermia/ hypothermia

ABCs

- Consider oxygen support via nonrebreather for adequate oxygenation
- Support adequate circulation
  - IV access
  - Fluid bolus (20ml/kg NS or LR) push pull system versus pump

ANTIBIOTICS

- Antibiotics within the first 60 minutes

ASSESSMENT AND REASSESSMENT

- Evaluate pulses, skin perfusion, mental status, urine output, blood pressure
- Escalation if necessary

SYSTOLIC BP GOALS	
<1 mo old	60 mm Hg
1 month to 10 years old	70 mm Hg + (2 x age in years)
>10 years old	90 mm Hg

ESCALATING CLINICAL CONCERNS

WHAT TO CALL	FOR WHOM	WHO COMES?
**Staff Assist**<br>**RED Button**	• Inpatients	Local response
**RRT x 2222**<br>(Rapid Response Team)	• Inpatients	15 minutes, PICU team
**CODE BLUE**<br>**BLUE Button or x 2222**	• Inpatients<br>• Dialysis<br>• Radiology	Large team, led by PICU
**CODE EARS x 2222**<br>(Emergency Ambulatory Response System)	• Outpatient clinics<br>• Visitors<br>• Staff	ED RN & tech with stretcher
**AIRWAY STAT**<br>**BLUE Button**	• PACU<br>• OR	Local Response
911	• Outpatient satellite clinics	EMS

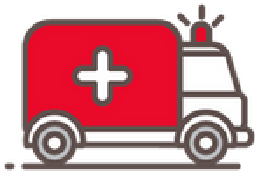

BEAR FRAMEWORK FOR COMMUNICATION

BOND → Establish a connection

- Make introductions
- Consider your body language
- Check-in on patient & caregiver well-being

ENGAGE → Build a partnership

- Active listening
- Ask questions
- Validate feelings
- Establish clear expectations

ASSESS → Evaluate partnership

- Clarify understanding of situation
- Managing expectations
- Self-reflection
- Frequent check-ins

REINFORCE → Strengthen & maintain partnership

- Summarize interaction
- Validate caregiver perspective
- Ensure everyone has the information necessary for next steps

REPEAT!

Remember - It takes 5 positive interactions for every difficult moment to build a positive relationship.

ESCALATING VERBAL & PHYSICAL VIOLENCE

WHAT TO CALL	FOR WHOM	WHO COMES?
**Social work early notification**	• **Parents/Caregivers**<br>• **Patients**	**Unit/Clinic Social Worker**
**SWIFT x2222** (Social Work Intervention with Families and Teams)	• **Parents**<br>• **Caregivers**	**Social Work + Security**
**BERT x 2222** (Behavioral Emergency Response Team)	• **Inpatient**	**Security + Psychiatry**
**Security x 2065**	• **Parents/Caregivers**<br>• **Patients**	**Security**

ADDITIONAL NOTES FOR SECURITY

- Security can be called to conduct a walkthrough of a certain room(space) if staff feel parents/visitors have the potential to escalate.
- Security **MUST** be called once a threat is made i.e. (parent on parent, parent on patient or parent on CNH staff) to investigate the situation.
- Security **MUST** be called whenever a physical altercation between (parent on parent, parent on patient, parent on staff or parent on guest) occurs.
- **Security/Chaplain or Security/Social Work** should be involved before parents receive any negative information regarding the patient treatment.
- Security should be considered (x2065) whenever a staff member feels unsafe performing their specific job duties.

PROMPTS TO CONSIDER

The prompts below are strategies we can use when families/caregivers appear upset, anxious, frustrated, etc. Please use the escalation resources above to support parents/caregivers, remembering the need to maintain a calm tone and body language.

DON'T SAY THAT →	SAY THIS INSTEAD...
"What do you need?"	"How can I help you right now?"<br><br>"How can I help you get through this difficult time?"
"I will have Security escort you out."	"I want to be able to help you with this. However, in order to move forward with this conversation, we all need to feel safe. What can I do so that we can have this conversation?"
"You need to calm down."	"This situation seems very frustrating. I/We are here to listen to you. Tell me more about xxxx."<br>"I hear how worried you are about xxxx. Tell me more about what you are worried about."<br>"Tell me what you need right now."<br>"What is the most important thing you need right now?<br><br>"What is the most important piece of information you need right now?"
"If you keep yelling at me, I'll call security."	"Tell me what you are upset about. Let's find a place to talk where you can have some privacy."<br>"I want to be able to focus on your concerns."<br>"Tell me what's happening. I hear and see you are upset."<br>"Tell me what is going on that has upset you."<br>"I want to have this conversation with you and when you yell, it is much harder for me to follow what you are saying."

OTHER STRATEGIES TO CONSIDER

LISTEN WITHOUT INTERRUPTION, LET THE PARENT SPEAK.

- This often leads to de-escalation and creates a space for the conversation to continue in a more productive and calm approach.

DON'T DEFEND AN ACTION DONE BY A COLLEAGUE THAT UPSET THE PARENT.

- Listen to the parent and then ask questions: *"What do you want to happen next? Where do we go from here? What is most important right now?"*
- Trying to defend an action by a staff member, even when the action was correct, can be interpreted as "not listening."
- If it is necessary to explain an action, it is better done when the parent is calm and has resolution.

FIND A "WIN"

- Can the team and parent come to a place where the parent gets something they are asking for or something they want?
- This strategy requires negotiation. The parent may not get their #1 ask, but the goal is to find something they are asking for that can be seen as a "win" by the parent!

FOCUS ON MOVING FORWARD

- Determine if the parent needs to speak with other team members based on the information provided.

WELL-BEING RESOURCES FOR SUPPORT

- CLICK HERE for a comprehensive list of employee resources (Go to Intranet >> Employee Services >> Benefits & Wellness)

Content by: Abigail Nolan MD & Heather Walsh PhD RN
Designed by: Ananya Datta CPNP-AC/PC, MPH RN
