# Supplementary material for: Simulation Addressing Verbal Escalation (SAVE): An Interprofessional Simulation for Pediatric Health Care Professionals
Source: MedEdPORTAL. 2026 Apr 15;22:11593. doi: 10.15766/mep_2374-8265.11593 (PMC13080524; doi:10.15766/mep_2374-8265.11593)
Supplement: Supplementary file 1 — Simulation Cases.docxSP Case.docxLearner Guide.pdfFacilitator Guide.docxTraining Slides.pptxTechnical Support Checklist.docxFlyer.pdfFeedback Survey.pdfFacilitator Debrief Worksheet.pdfPresurvey.pdf [file mep_2374-8265.11593-s001.zip › D. Facilitator Guide.docx]

**Simulation Addressing Verbal Escalation (SAVE) Training Rev 10-21-24**

**Facilitator Guide**

Spoken Items in *Italics*

# Session Details

# Four 60 min sessions per scheduled event

# 30 min prior: setup/arrival

# Hours 1-4: 1-hour sessions x4

# 30 minutes after: breakdown

# 2x/week, 1 morning session, 1 afternoon session; days will rotate

# Up to 14 learners (4 APPs/Physicians, 6 RNs, 4 Other); will cancel if < 3 learners

# Personnel

# 2 Primary Facilitators (1 from sim team)

# 1 Backup Facilitator

# 1 Simulation Operations Specialist

# 1-2 SPs

# Session Agenda

| Sign-In/Orientation/Structured Pre-Brief | 10 min (Begin no later than 5 min after hour) |
| --- | --- |
| Scenario 1 – Acute Infant Sepsis with Concerned Parent | 10 min |
| Debriefing 1 – Acute Infant Sepsis with Concerned Parent | 12 min |
| Scenario 2 – Worsening Sepsis in Toddler with Escalating Parent | 10 min (Begin no later than 35 min after hour) |
| Debriefing 2 – Worsening Sepsis in Toddler with Escalating Parent | 15 minutes |
| Wrap-Up | 3 minutes |

**Sign-In/Orientation/Structured Pre-Brief**

**Slide 1 – Pre-Session Evaluation (as learners arrive, periodically draw attention to the QR Code for the Optional Research Study; each survey should take less than 3 minutes)**

As people filter in, Facilitator 1: *We are evaluating the effectiveness of this training through a series of optional surveys that ask questions regarding your thoughts on verbal violence in the workplace. While participation in the SAVE Training is required, your participation in this research is optional. If you wish to participate in this optional research, please use this QR code (on slide/paper). The survey takes less than 3 minutes to complete.*

**Slide 2 – Do not need to read attestation verbatim; let learners know we are offering 1 hour of CE credits**

At no later than 5 min past the hour, start

Facilitator 1: *Thank you for attending today.* (State name and role at Children’s National). *I will be one of your facilitators for today’s training*.

Facilitator 2: (State name and role at Children’s National). *I will be your second facilitator for this training.*

Tech Support: (State name and role at Children’s National). *I will be providing support during the simulation.*

Facilitator 1: *Finally, we’d like to introduce our Standardized Participant, or SP for short, who will portray the parent in today’s scenarios. The SP is highly trained in these scenarios and will respond to you as appropriate for each scenario.*

**Slide 3 – Agenda (do not need to read agenda verbatim, leave slide up as you continue script)**

Facilitator 2: *Our training today will emphasize the core values of Compassion, Commitment and Connection. We will use scenarios involving a decompensating patient and will address the complexities of human emotions and behaviors that we face when interacting with patients, families, and visitors. Our objective is to enhance your clinical competency in managing interactions with others. Our core values will serve as a foundation in our approach to empowering you to provide exceptional care to our patients and families in a safe and inclusive environment.*

Facilitator 1: *Before we begin, we would like to take a moment to ask you to introduce yourselves to each other. Please share your name and role at Children’s National. For our icebreaker today please share a stressor you’ve observed/ encountered in your experience working with families here.*

**Slide 4 – Ground Rules**

Facilitator 2: *Let’s review the ground rules of today’s session:*

- *To maintain an equitable learning environment, we will use first names to address each other and encourage you to do the same.*
- *We believe that everyone participating in this simulation is intelligent, cares about doing their best, and wants to improve.*
- *We ask that you maintain confidentiality of this group learning experience… what we say or do in simulation, stays here. We understand that the manikins and SPs are not real patients and parents, and the environment may not be your actual clinical environment, but we encourage you to act as you normally would in a real clinical situation.*
- *The simulations today are meant to be formative, to help us improve. This is not a “test”. We aim to further develop your knowledge, skills, and abilities to improve clinical responses and interpersonal interactions and the delivery of patient-family centered care.*
- *We don’t expect you to perform perfectly. In fact, we view mistakes as learning opportunities for the whole group.*
- *We aim to create a safe learning environment and acknowledge that you may have experienced situations with verbal escalation in your clinical practice.*

**Slide 5 – Scenario 1**

Facilitator 1: *While we encourage you to apply this learning and to share what you learned with others, we’d ask you to not discuss the performance of others and the specific scenarios with anyone. A tremendous amount of effort goes into developing scenarios and sharing the specifics could diminish the learning for future participants.*

*Just as in simulations involving a deteriorating patient where you might call a rapid response or a code blue, in these scenarios with verbal escalation, you can access hospital resources such as calling your unit/ department social worker or a code SWIFT.*

Facilitator 2: *Some of you may not be familiar with simulation so I would like to first orient you to the environment.*

- Orient participants to the space, equipment/supplies, simulator, patient situation/setting, limitations, realism/fidelity, SP.
- Identify resources
- We will provide information only when we observe the actions such as assessment of cap refill, skin color, or neurological state changes
- Orient participants on how they will work together in the scenario (Teams A and B – one per scenario – if more than 6-8 learners in a session)
- Allow learners to ask any questions before beginning

**Scenario 1**

Summary (Read to Group)

*It is* *change of shift and you have just received a brief handoff: Simon Jones is an 11mo M with acute viral bronchiolitis who has been in the hospital for 24 hours and is currently on 2L nasal cannula. You are on the acute care floor (could be the ED depending on the learners). You are in the unit hallway as the scenario begins.*

Scenario starts with caregiver calling for the nurse; do not read following information – it is provided here for your reference.

| **Expected Simulation Run Time:** 10 min **Debriefing Time:** 12 min  **Age:** 11mo M  **Weight:** 8 kg  **HPI:**  Simon Jones is an 11mo M with acute viral bronchiolitis who has been in the hospital for 24 hours and is currently on 2L nasal cannula. He had been doing well until recently when caregiver calls the nurse because he is less responsive. The team needs to identify sepsis and initiate treatment. Once 20 cc/kg NS has been given to the patient, vital signs improve, and case stops. |
| --- |

Scenario Progression

| **Segment/ timing:** | **Manikin Actions** | **Interventions learners are expected to:** | **May use the following cues:** |
| --- | --- | --- | --- |
| **Initial assessment**:  (time in segment)  3 minutes | **VS:**  T 35 C rectal  HR 200, sinus  RR 70  BP 75/35 (48)  O2 Sat 95%  **PE:** moaning, minimal movement  cap refill 4-5 sec, mottling  Subcostal retractions  Abdominal exam: soft, non-distended  **Monitor:** | **Assessment:**   - ABC/ Pediatric assessment triangle.   **Actions:**   - Assesses patient - Identifies sepsis - Asks for fluids - Ask for antibiotics - Asks for fever control (acetaminophen, ibuprofen) - Start oxygen to help perfusion - May assign a team member to communicate with Caregiver | Can give PE description when asked  Can give labs when asked  WBC 18 Hgb 10 HCT 30 Plt 300 Neutrophils 70% Bands 5%  When asked: Normal CXR  SP actions:   - Caregiver can give background and history as detailed on SP form - Caregiver asks pertinent questions like, “Is Simon going to be okay?” |
| **Decompensation:**  (time in segment)  5 minutes | **VS:**  T 35  HR 190  RR 70  BP 60/25 (35)  O2 Sat 92%  **PE:**  Lethargic, responds to painful stimulus  Still tachypneic/tachycardic  **Monitor:** | **Assessment:**   - Reevaluate ABCs   **Actions:**   - Identifies shock like state - Prioritizes fluids with push pull - If on acute care, calls for ICU (rapid response) - Identifies escalating parent and asks for social worker, child life, or the chaplain - If not already done, may assign a team member to help gather information | SP actions:   - As more people arrive, caregiver can get more concerned, ask more questions - Caregiver should express a lack of understanding of plan |
| **Case resolution/or**  **clinical improvement**  (time in segment)  **5 minutes**  **Resolves when push/pull finishes** | **VS:**  HR 150s  RR 40s  BP 80/60  O2 Sat 100%  **PE:**  Improved cap refill  Improved respiratory status  **Monitor:** | **Assessment:**   - Reevaluate ABCs   **Actions:**  Discusses sepsis with team |  |

Debriefing

Initial Questions:

- *How did that feel?*
- *Can someone please summarize the case for the group?*
- *What went well?*
- *How did you perform as a team?*

Feedback from SP

- *How did you feel?*
- *What did the team do well?*
- *What could have been done better?*

Targeted Questions:

- *Was there a shared mental model?*
  - *Among the team, with the parent?*
- *Were team roles & responsibilities assigned?*

**Slide 6 – BEAR Framework**

*We’d like to reinforce using the BEAR framework for communicating with patients and families.*


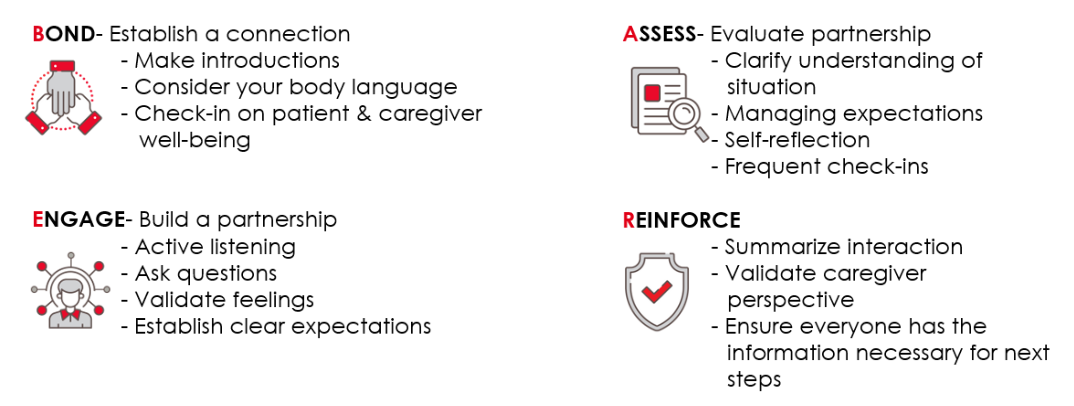


**Slide 7 – Sepsis Management**

Takeaways (Do not need to read verbatim or spend time here if most steps were performed)

- Resuscitation:
  - Recognize sepsis, verbalize sepsis
  - Immediate steps:
    - Ensure adequate airway, oxygenation, ventilation
    - Support adequate circulation
      - IV access
      - Fluid bolus
        - As fast as possible
        - Push pull system versus pump
      - Target HR and perfusion parameters
      - Temperature control
      - Antibiotics within the first 60 minutes, or per protocol
  - Assessment and re-assessment are important
    - Evaluate pulses, skin perfusion, mental status, urine output, blood pressure
    - Systolic BP goals
      - <1 month of age – 60 mmHg
      - 1 month to 10 years of age – 70 mmHg + [2 x age in years]
      - 10 years of age and older – 90 mmHg

**Slide 8 – Clinical Escalation**

*We wanted to remind you of available resources for clinical deterioration and encourage you to escalate care at any time.*

**Slide 9 – Scenario 2**

**Scenario 2**

Summary (Read to Group)

*Simon has returned to the hospital now as a 2-year-old admitted with flu and pneumonia who is tachycardic, tachypneic, and febrile. Again, it is change of shift and you have just received a brief handoff. You are in the unit hallway as the scenario begins.*

Scenario begins with caregiver coming out of the room calling for help. do not read following information – it is provided here for your reference.

| **Expected Simulation Run Time:** 10 min **Debriefing Time:** 15 min  **Age:** 2 yo M  **Weight:** 12 kg  **HPI:**  **Acute care or ICU/ED: Simon** is a 2 yo M with flu and pneumonia who is now tachycardic, tachypneic and febrile. Caregiver is on edge because he has been in the ED for 19 hours waiting on a floor bed and was just admitted to acute care unit. Caregiver and patient have both not slept in 2 days. The caregiver gets quite agitated as their son decompensates requiring fluid resuscitation for sepsis. |
| --- |

### Scenario Progression

| **Segment/timing:** | **Manikin Actions** | **Interventions learners are expected to:** | **May use the following cues:** |
| --- | --- | --- | --- |
| **Initial assessment**:  (time in segment)  3 minutes | **VS:**  T 39 C  HR 160  RR 50  BP 75/35 (48)  O2 Sat 95% on 2 L NC  **PE:** moaning, minimal movement  Prolonged cap refill  Subcostal retractions  Abdomen: soft, nontender  **Monitor:** | **Assessment:**   - ABC/ Pediatric assessment triangle.   **Actions:**   - Assesses patient - Identifies sepsis - Establishes 2 IV access - Asks for fluids - Ask for antibiotics - Asks for fever control (acetaminophen, ibuprofen) - Start oxygen support | Can give PE description when asked  Can give labs when asked  WBC 18 Hgb 10 HCT 30 Plt 300 Neutrophils 70% Bands 5%  When asked: CXR with right focal consolidation  Caregiver is irritated/visibly annoyed |
| **Decompensation:**  (time in segment)  5 minutes | **VS:**  T 39  HR 170  RR 70  BP 60/25 (35)  O_2_ Sat 88% (unless placed on NRB then 94%)  **PE:**  Lethargic, responds to painful stimulus  Still tachypneic/tachycardic  Delayed cap refill | **Assessment:**   - Reevaluate ABCs   **Actions:**   - Identifies shock like state - Prioritizes fluids with push pull - Calls for help depending on setting (Acute care calls RRT versus code, ED calls medical alert (+/- PICU), ICUs call staff assist - Call SW or identify staff member to support | Caregiver exhibits verbal escalation, maneuvers to get to the patient, yelling and stating things like “you are hurting my child”  Caregiver may threaten to take Simon AMA |
| **Case resolution/or**  **clinical improvement**  (time in segment)  **5 minutes**  **Resolves when one NS push/pull bolus finishes** | **VS:**  HR 150s  RR 40s  BP 80/60  O2 Sat 100%  **PE:**  Improved cap refill  Improved respiratory status | **Assessment:**   - Reevaluate ABCs   **Actions:**  Discusses sepsis with team |  |

**Slide 10 – BEAR Framework**

Debriefing

Initial Questions:

- *How did that feel?*
- *Can someone please summarize the case for the group?*
- *What went well?*
- *What would you improve upon?*
- *How did you perform as a team?*

Feedback from SP

- - *How did you feel as the caregiver?*
  - *What did the team do well?*
  - *What could have been improved?*
- *What else could have been going on with the caregiver?*
- *What resources are available when dealing with a parent who is verbally escalating?*

*(What resources are available on nights, weekends?)*

- *How might you have responded differently to the caregivers if…*
  - - *Presented as a different gender or stature?*
    - *Presented as different race?*
    - *Spoke in a different manner (volume, cadence, language, cursing)?*
    - *2 caregivers were present?*
    - *Was not a native English speaker?*

Targeted Questions (optional, if time):

- - *How might the family’s previous experiences with healthcare, such as the first scenario, inform their current actions/choices/words?*
- *How do our implicit biases affect these interactions?*

Takeaways

*Again, we’d encourage you to use the BEAR framework when communicating with patients and families.*


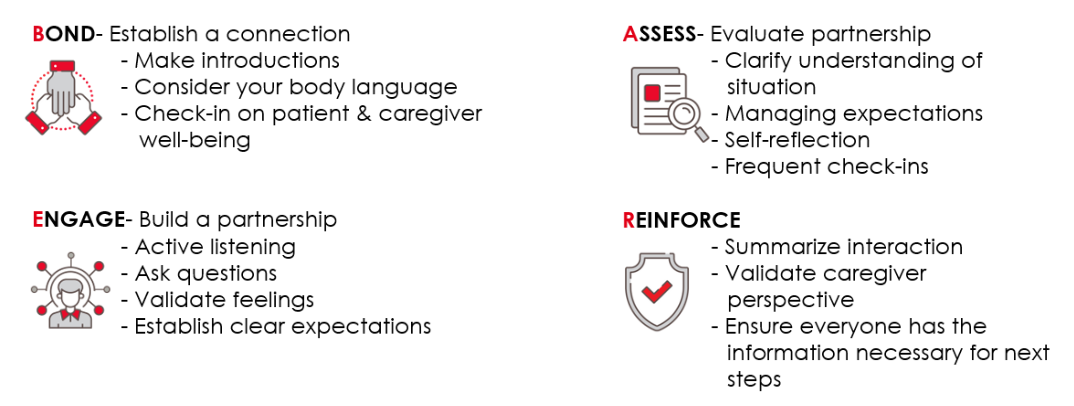


- De-escalation techniques
  - Redirection
    - focus on patient
    - redirection, for example, tell me more about what’s going on
  - Proximity/Spatial awareness of the family member
    - Placing them in an appropriate place in the room
    - Positioning a person close to them
  - Behavior Management
    - Directly stating what is appropriate and what is not
    - Setting standards and boundaries

**Slide 11 – Escalation of Verbal and Physical Threats/Violence**

- - Resources
    - Patient Support: Child Life, Chaplaincy, SW
    - Unit-based Leadership: Attending, Charge Nurse, Educators, Nurse Managers
    - Hospital-based Leadership: AD, Ombudsman
  - Safety Event Reporting System

**Slide 12 – Safety Event Reporting**

**Wrap-Up**

**Slide 13 – Training Feedback and Optional Research**

Facilitator 1*: Please use this QR code to complete an anonymous feedback survey of this training. After you submit, you will be redirected to an optional survey that asks questions regarding your thoughts on verbal violence in the workplace. While participation in the SAVE Training was required, your participation in this second research survey is optional.*

Facilitator 2*: As you complete the surveys, please share one thing that you learned from this scenario, and/or how you plan to implement changes in your practice.*

**Slide 14 – CE Credit**

Facilitator 2: We are offering 1 hour of continuing education credit for this training. Please text this code to this phone number to claim CE credit. You may also enter this code through the CE website. Thank you for your attendance and participation today! We appreciate the expertise the SPs bring to the scenarios and would like to thank them for making this training impactful.

**Slides 15 – Resource QR Codes**

Facilitator 1: We hope that you will be able to utilize some of the tools provided today to provide high-level care for our patients and their families. The information provided today is also available on the Intranet. Also included are resources to help support you as employees. You can scan these QR codes for resources on Employee Wellbeing and a compilation of resources related to this training.

**FACILITATORS/TECH SUPPORT – Ensure Facilitator Debrief Worksheet completed**
