# Supplementary material for: Simulation Addressing Verbal Escalation (SAVE): An Interprofessional Simulation for Pediatric Health Care Professionals
Source: MedEdPORTAL. 2026 Apr 15;22:11593. doi: 10.15766/mep_2374-8265.11593 (PMC13080524; doi:10.15766/mep_2374-8265.11593)

## Slide 1
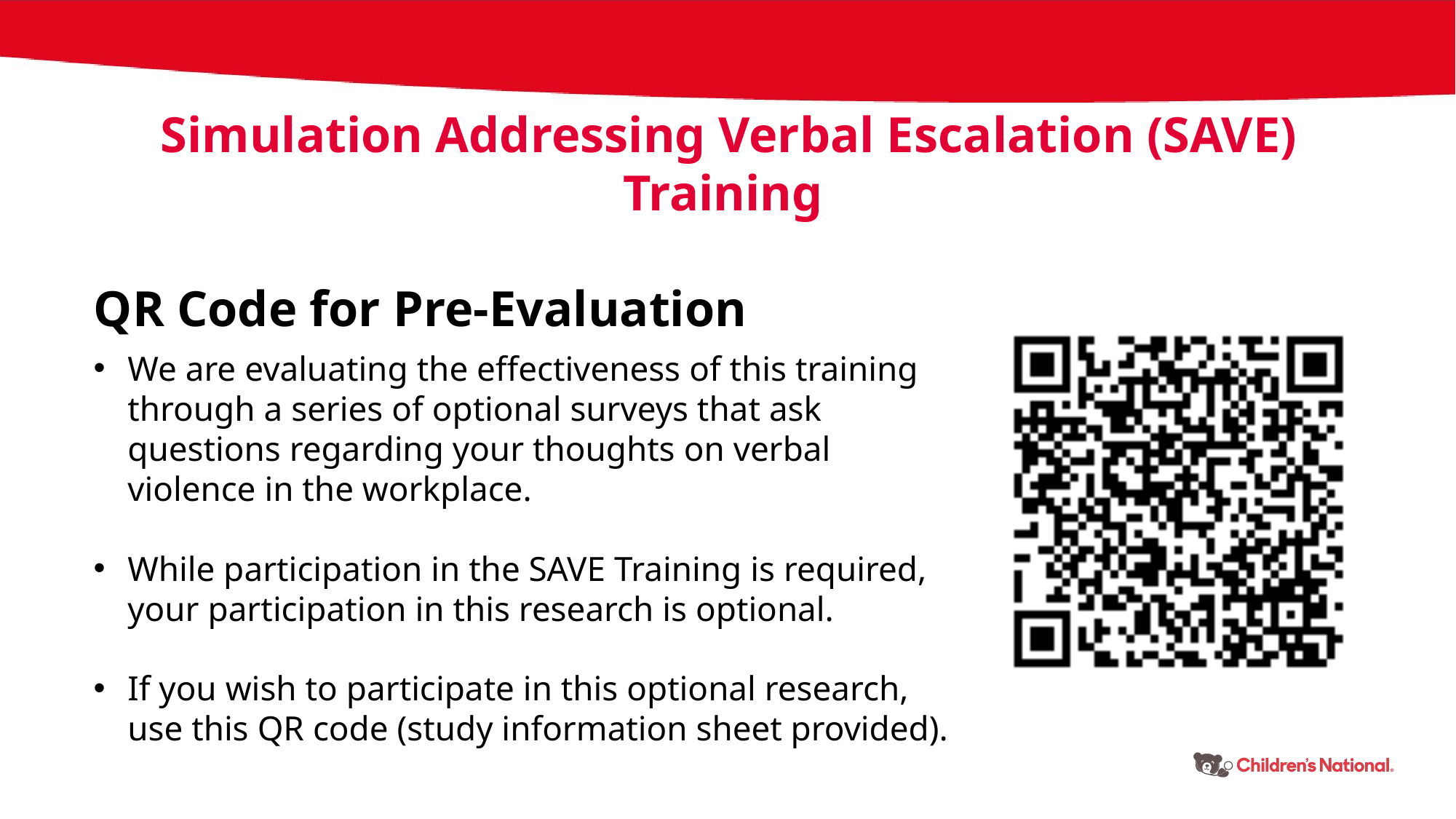

Simulation Addressing Verbal Escalation (SAVE) Training
QR Code for Pre-Evaluation
We are evaluating the effectiveness of this training through a series of optional surveys that ask questions regarding your thoughts on verbal violence in the workplace.
While participation in the SAVE Training is required, your participation in this research is optional.
If you wish to participate in this optional research, use this QR code (study information sheet provided).

## Slide 2
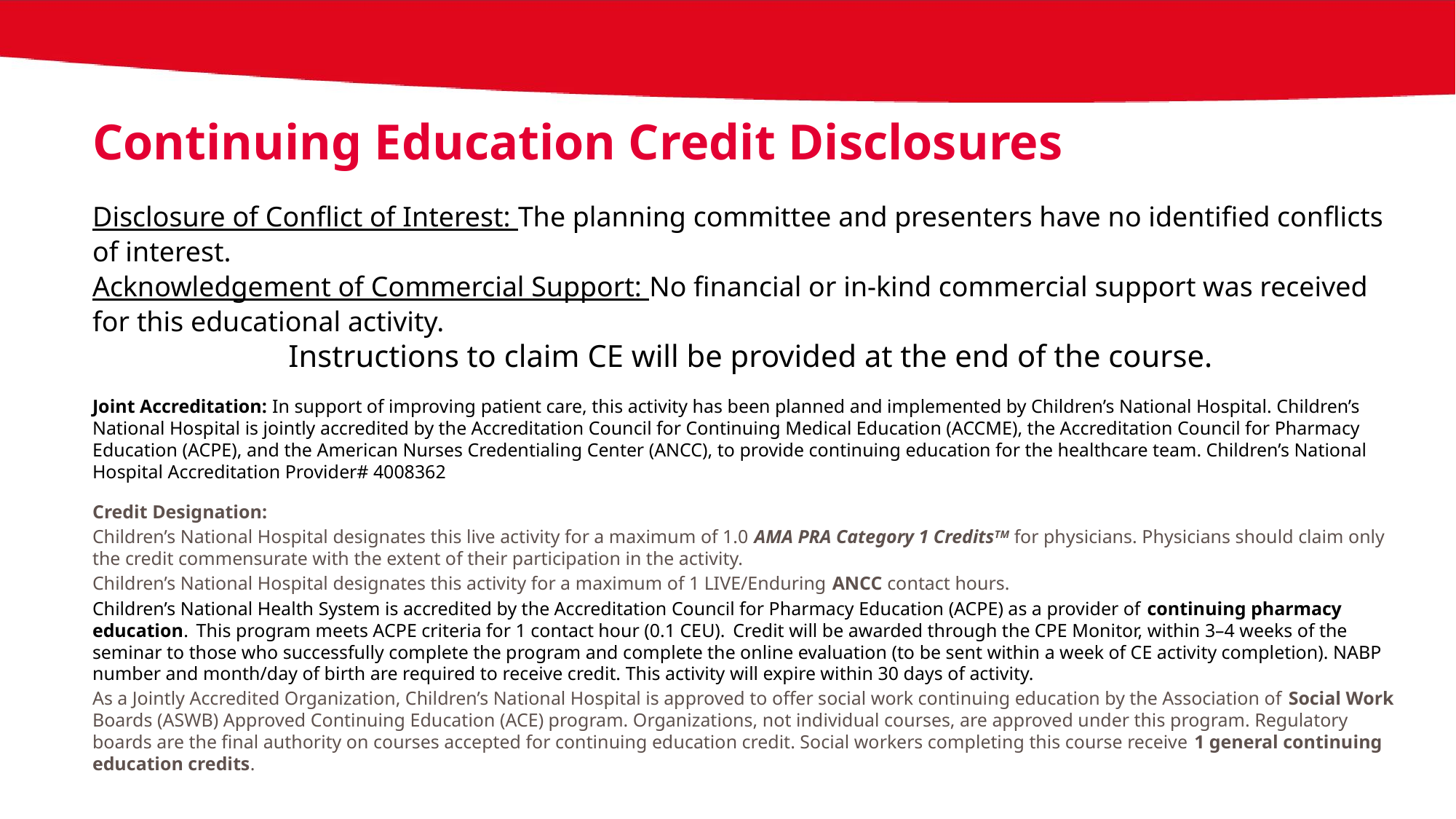

Continuing Education Credit Disclosures
Disclosure of Conflict of Interest: The planning committee and presenters have no identified conflicts of interest.
Acknowledgement of Commercial Support: No financial or in-kind commercial support was received for this educational activity.
Instructions to claim CE will be provided at the end of the course.
Joint Accreditation: In support of improving patient care, this activity has been planned and implemented by Children’s National Hospital. Children’s National Hospital is jointly accredited by the Accreditation Council for Continuing Medical Education (ACCME), the Accreditation Council for Pharmacy Education (ACPE), and the American Nurses Credentialing Center (ANCC), to provide continuing education for the healthcare team. Children’s National Hospital Accreditation Provider# 4008362
Credit Designation:
Children’s National Hospital designates this live activity for a maximum of 1.0 AMA PRA Category 1 CreditsTM for physicians. Physicians should claim only the credit commensurate with the extent of their participation in the activity.
Children’s National Hospital designates this activity for a maximum of 1 LIVE/Enduring ANCC contact hours.
Children’s National Health System is accredited by the Accreditation Council for Pharmacy Education (ACPE) as a provider of continuing pharmacy education.  This program meets ACPE criteria for 1 contact hour (0.1 CEU).  Credit will be awarded through the CPE Monitor, within 3–4 weeks of the seminar to those who successfully complete the program and complete the online evaluation (to be sent within a week of CE activity completion). NABP number and month/day of birth are required to receive credit. This activity will expire within 30 days of activity.
As a Jointly Accredited Organization, Children’s National Hospital is approved to offer social work continuing education by the Association of Social Work Boards (ASWB) Approved Continuing Education (ACE) program. Organizations, not individual courses, are approved under this program. Regulatory boards are the final authority on courses accepted for continuing education credit. Social workers completing this course receive 1 general continuing education credits.

## Slide 3
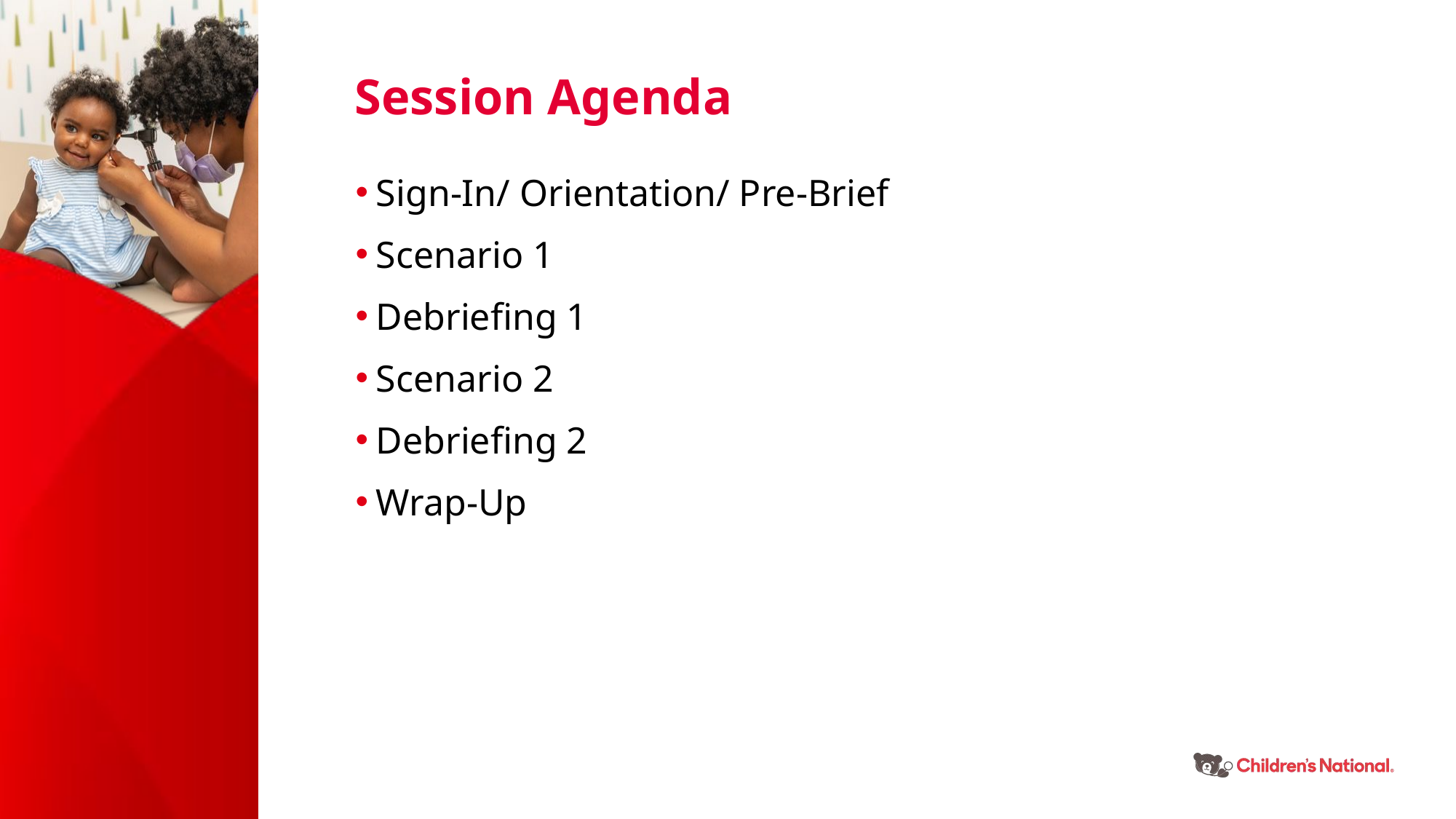

Session Agenda
Sign-In/ Orientation/ Pre-Brief
Scenario 1
Debriefing 1
Scenario 2
Debriefing 2
Wrap-Up

## Slide 4
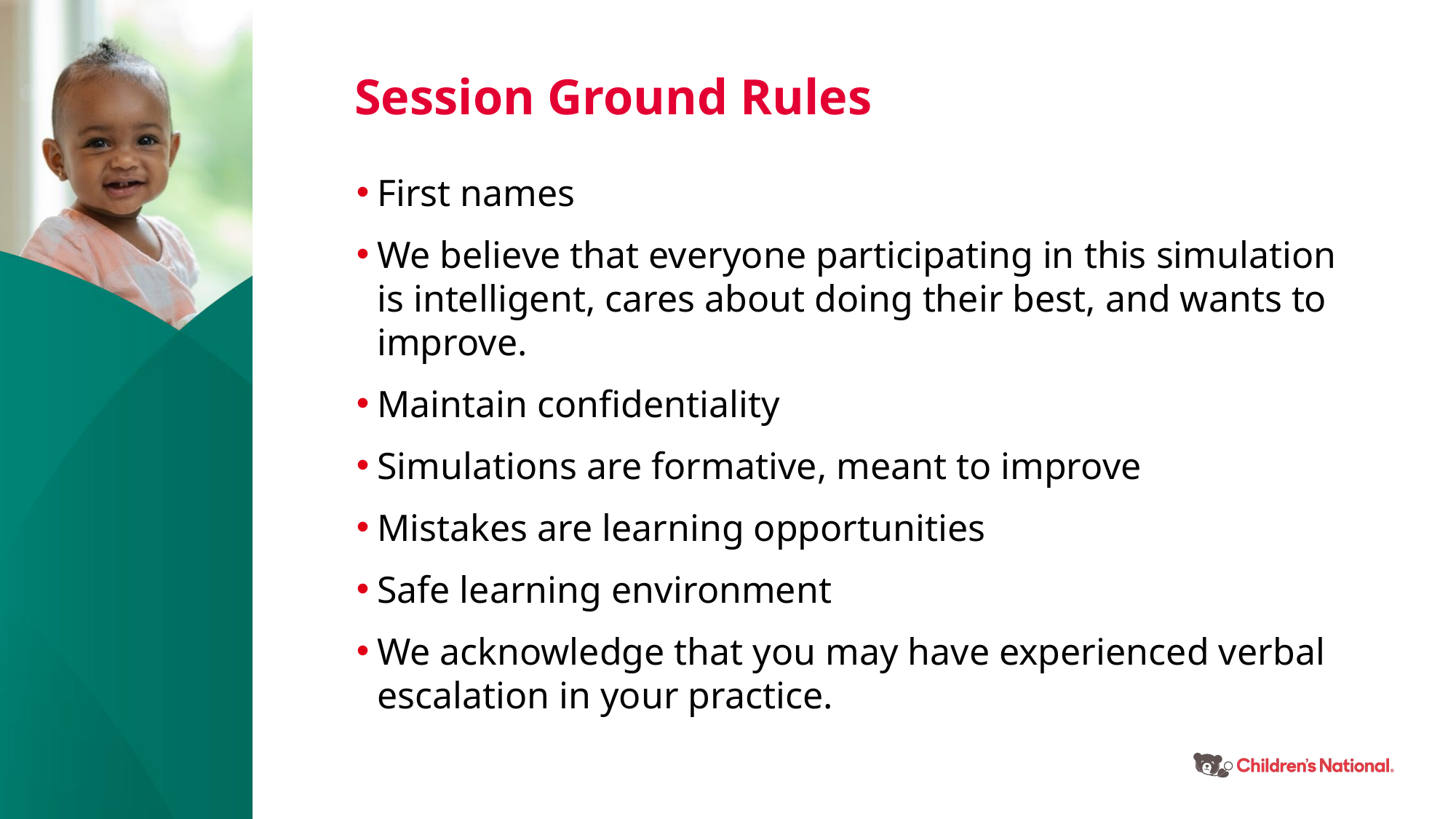

Session Ground Rules
First names
We believe that everyone participating in this simulation is intelligent, cares about doing their best, and wants to improve.
Maintain confidentiality
Simulations are formative, meant to improve
Mistakes are learning opportunities
Safe learning environment
We acknowledge that you may have experienced verbal escalation in your practice.

## Slide 5
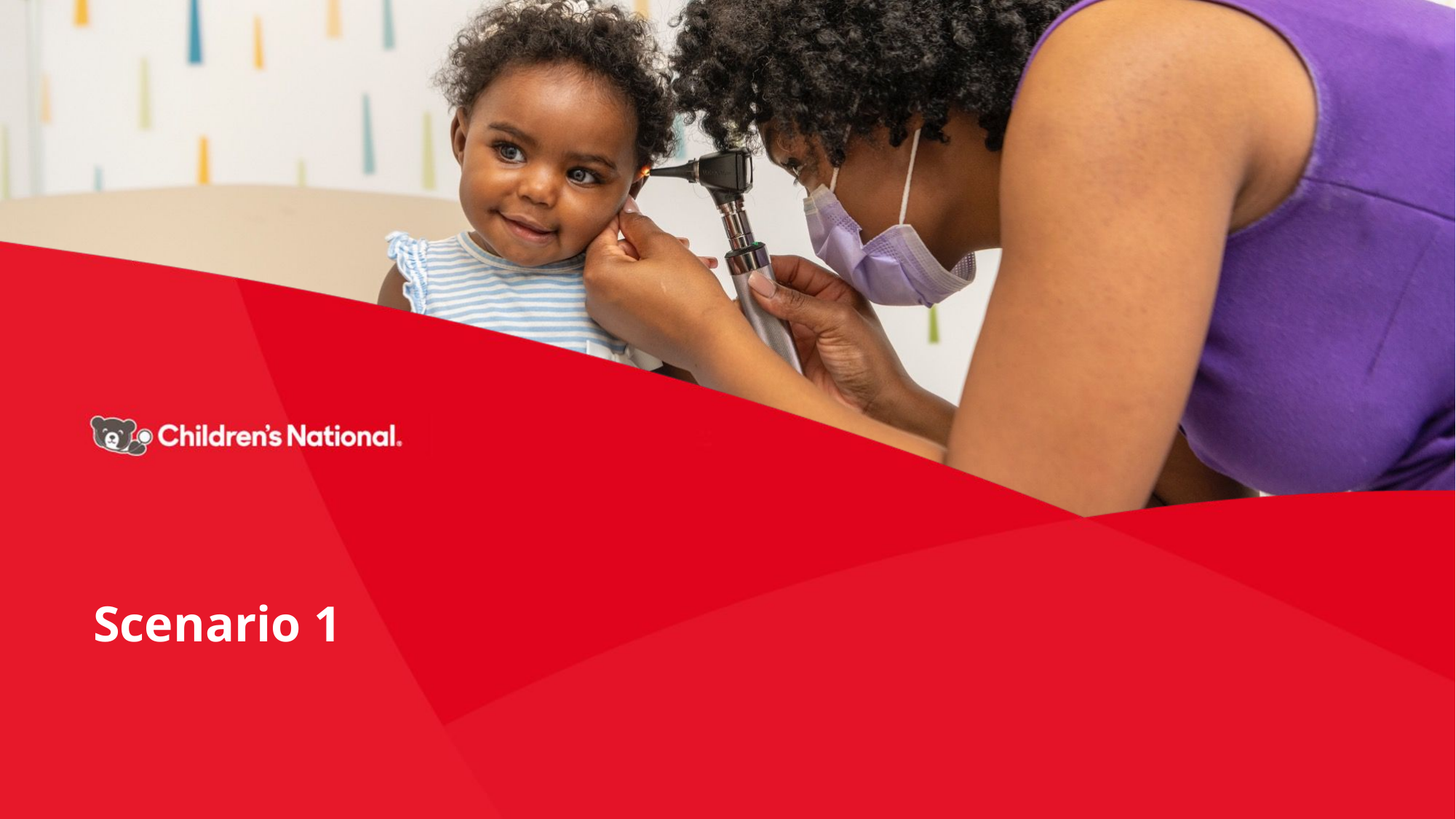

Scenario 1

## Slide 6
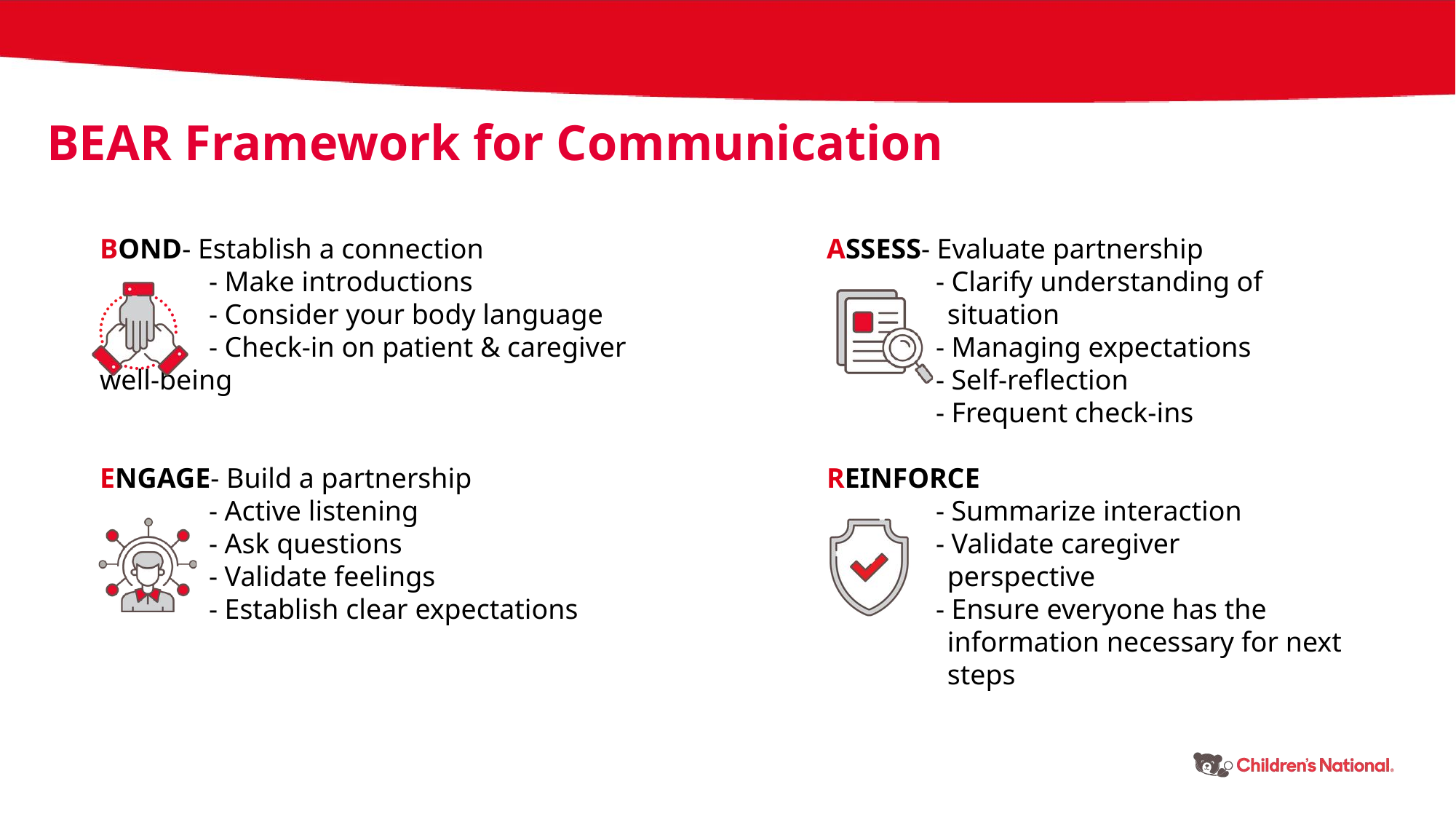

BEAR Framework for Communication
BOND- Establish a connection
	- Make introductions
	- Consider your body language
	- Check-in on patient & caregiver 	 well-being
ENGAGE- Build a partnership
	- Active listening
	- Ask questions
	- Validate feelings
	- Establish clear expectations
ASSESS- Evaluate partnership
	- Clarify understanding of
 situation
	- Managing expectations
	- Self-reflection
	- Frequent check-ins
REINFORCE
	- Summarize interaction
	- Validate caregiver
 perspective
	- Ensure everyone has the
 information necessary for next
 steps

## Slide 7
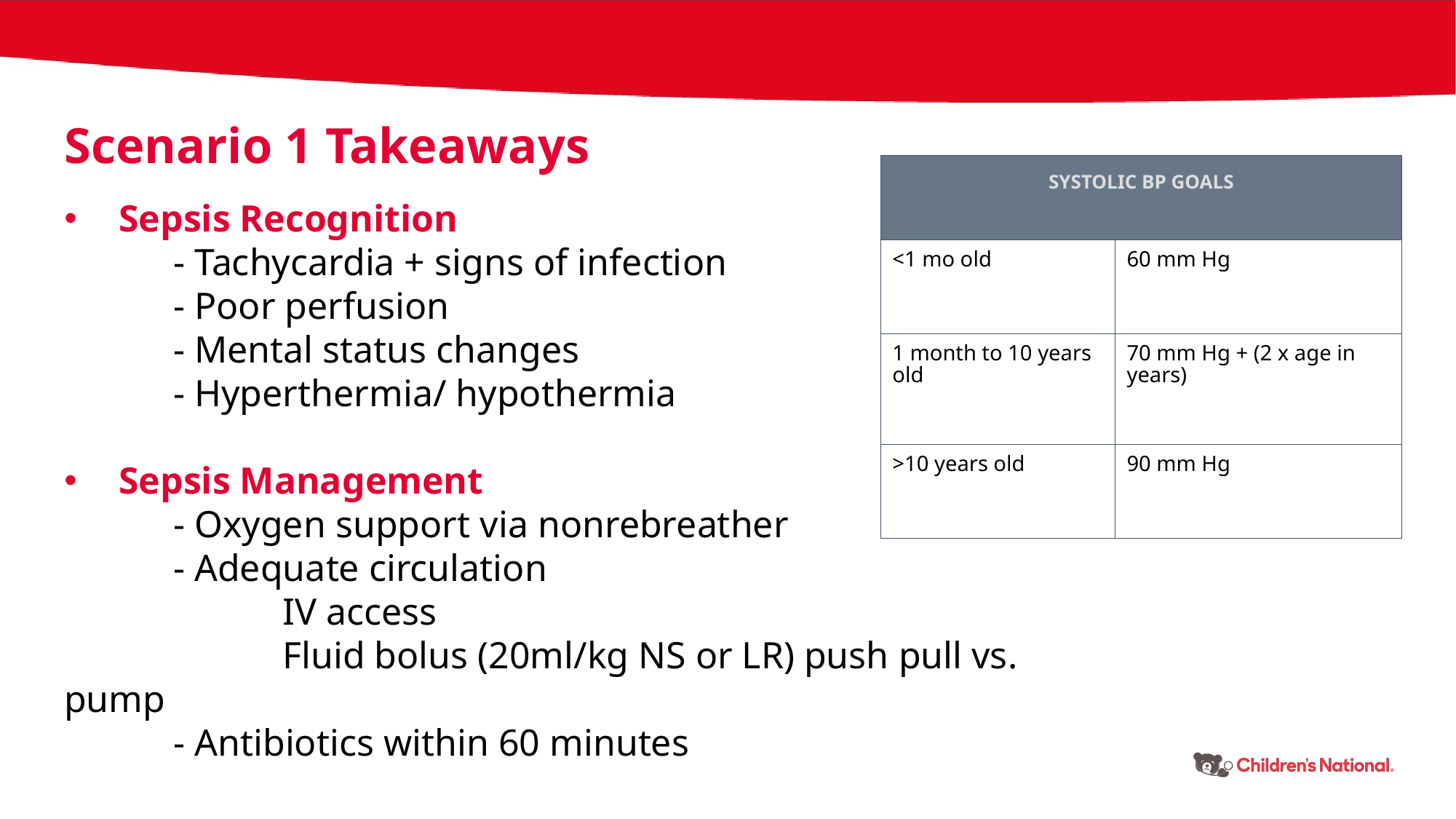

Scenario 1 Takeaways
| SYSTOLIC BP GOALS | SYSTOLIC BP GOALS |
| --- | --- |
| <1 mo old | 60 mm Hg |
| 1 month to 10 years old | 70 mm Hg + (2 x age in years) |
| >10 years old | 90 mm Hg |
Sepsis Recognition
	- Tachycardia + signs of infection
	- Poor perfusion
	- Mental status changes
	- Hyperthermia/ hypothermia
Sepsis Management
	- Oxygen support via nonrebreather
	- Adequate circulation
		IV access
 		Fluid bolus (20ml/kg NS or LR) push pull vs. pump
	- Antibiotics within 60 minutes

## Slide 8
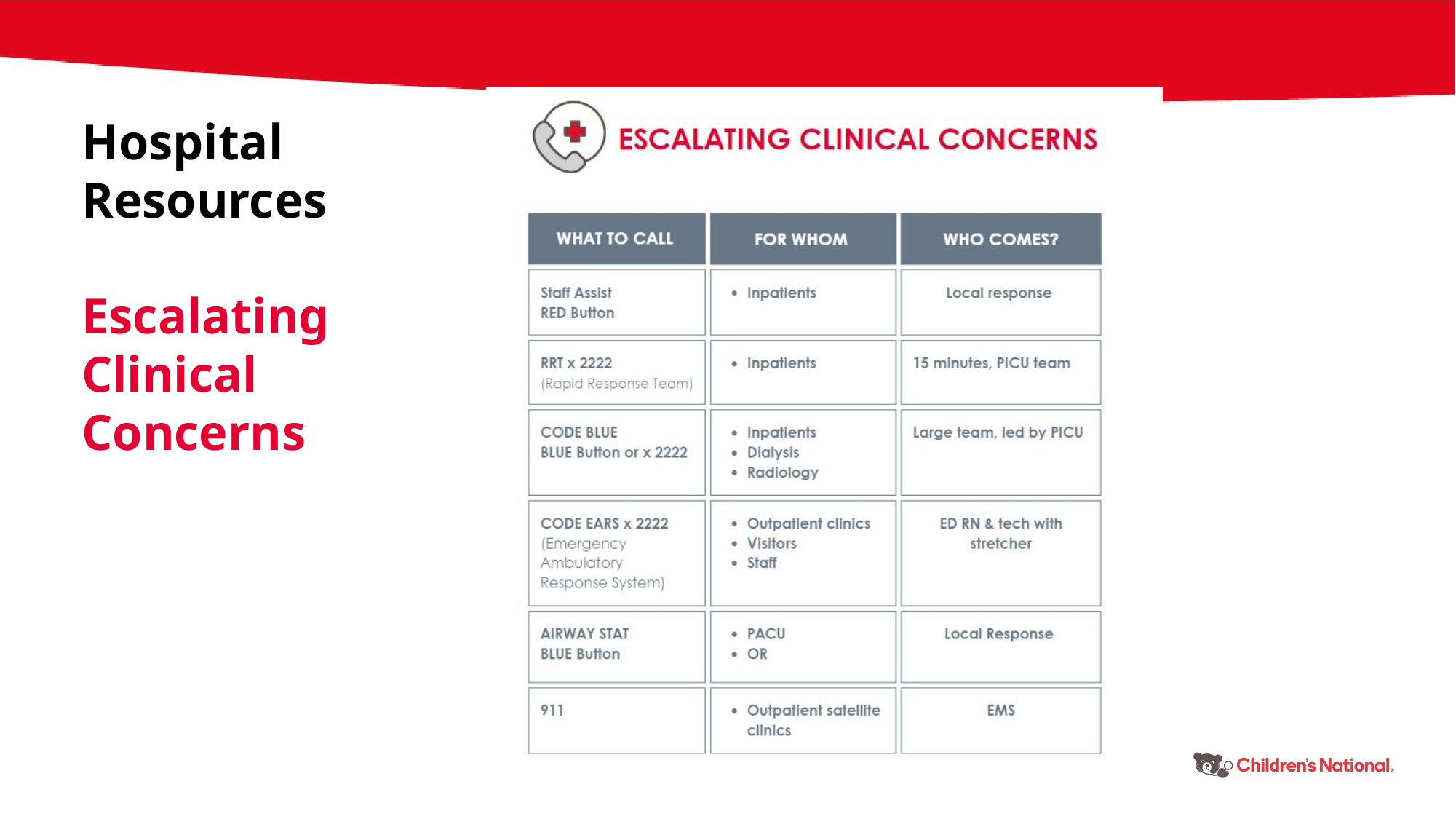

Hospital
Resources
Escalating
Clinical
Concerns

## Slide 9
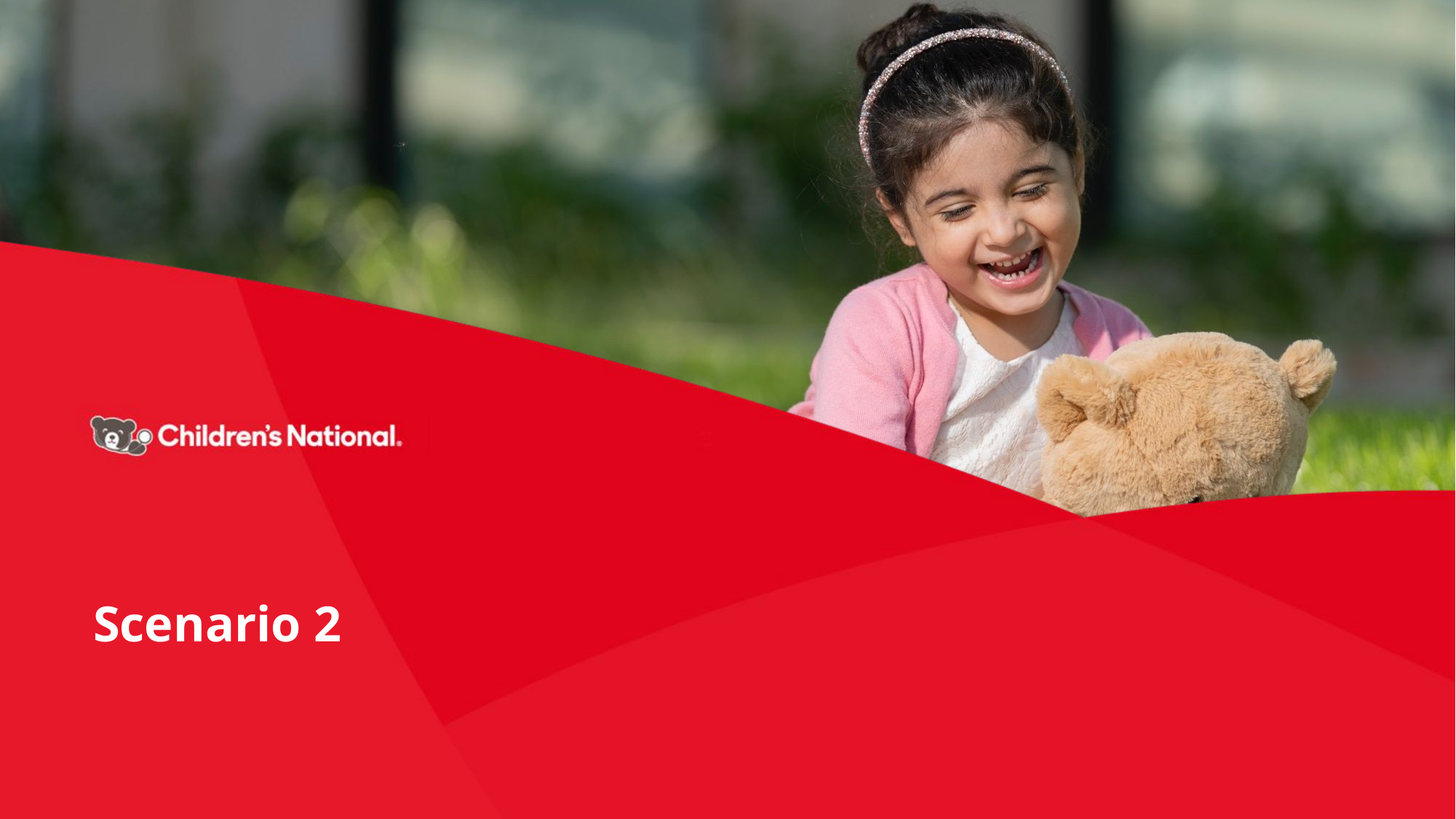

Scenario 2

## Slide 10
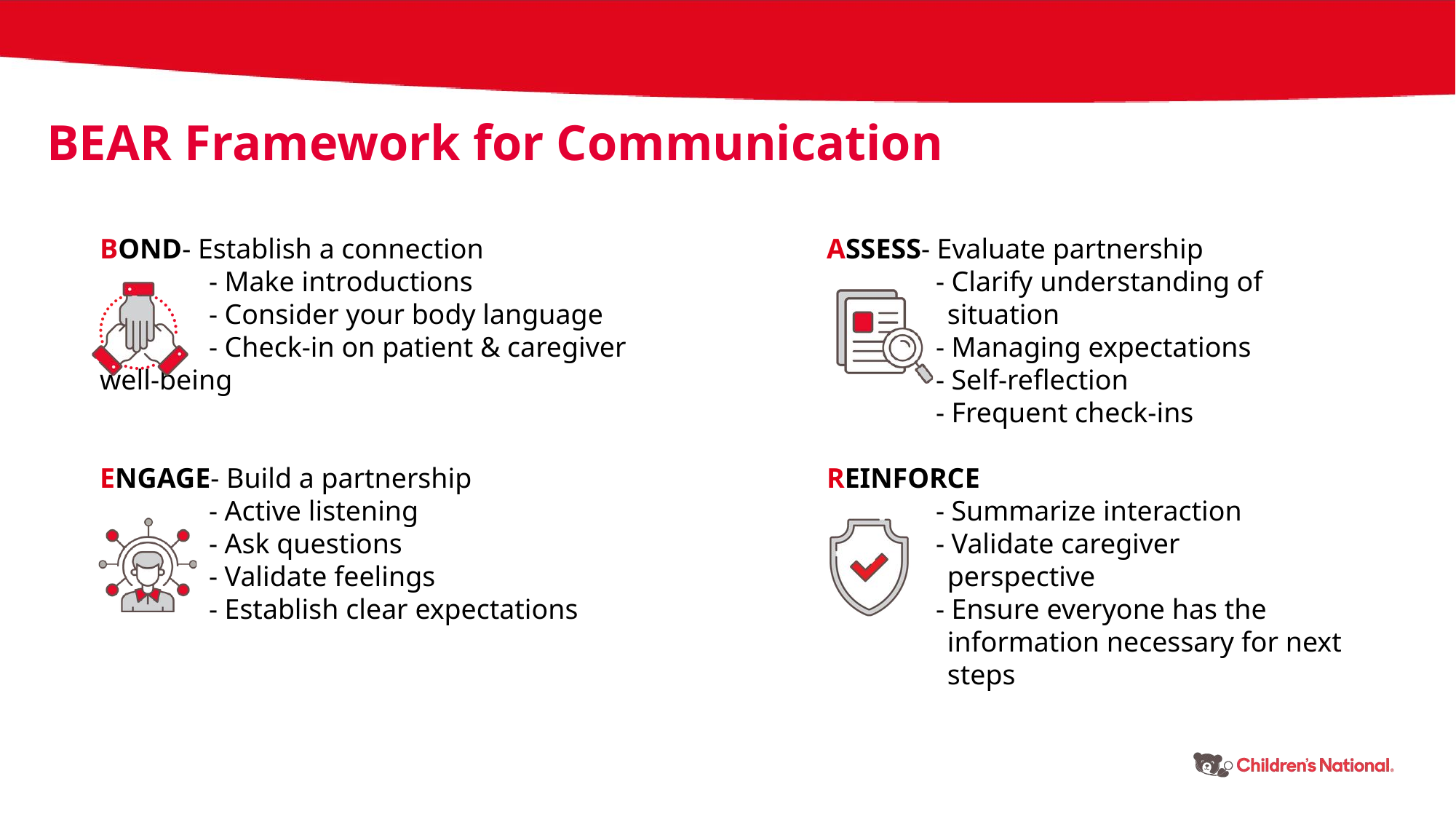

BEAR Framework for Communication
BOND- Establish a connection
	- Make introductions
	- Consider your body language
	- Check-in on patient & caregiver 	 well-being
ENGAGE- Build a partnership
	- Active listening
	- Ask questions
	- Validate feelings
	- Establish clear expectations
ASSESS- Evaluate partnership
	- Clarify understanding of
 situation
	- Managing expectations
	- Self-reflection
	- Frequent check-ins
REINFORCE
	- Summarize interaction
	- Validate caregiver
 perspective
	- Ensure everyone has the
 information necessary for next
 steps

## Slide 11
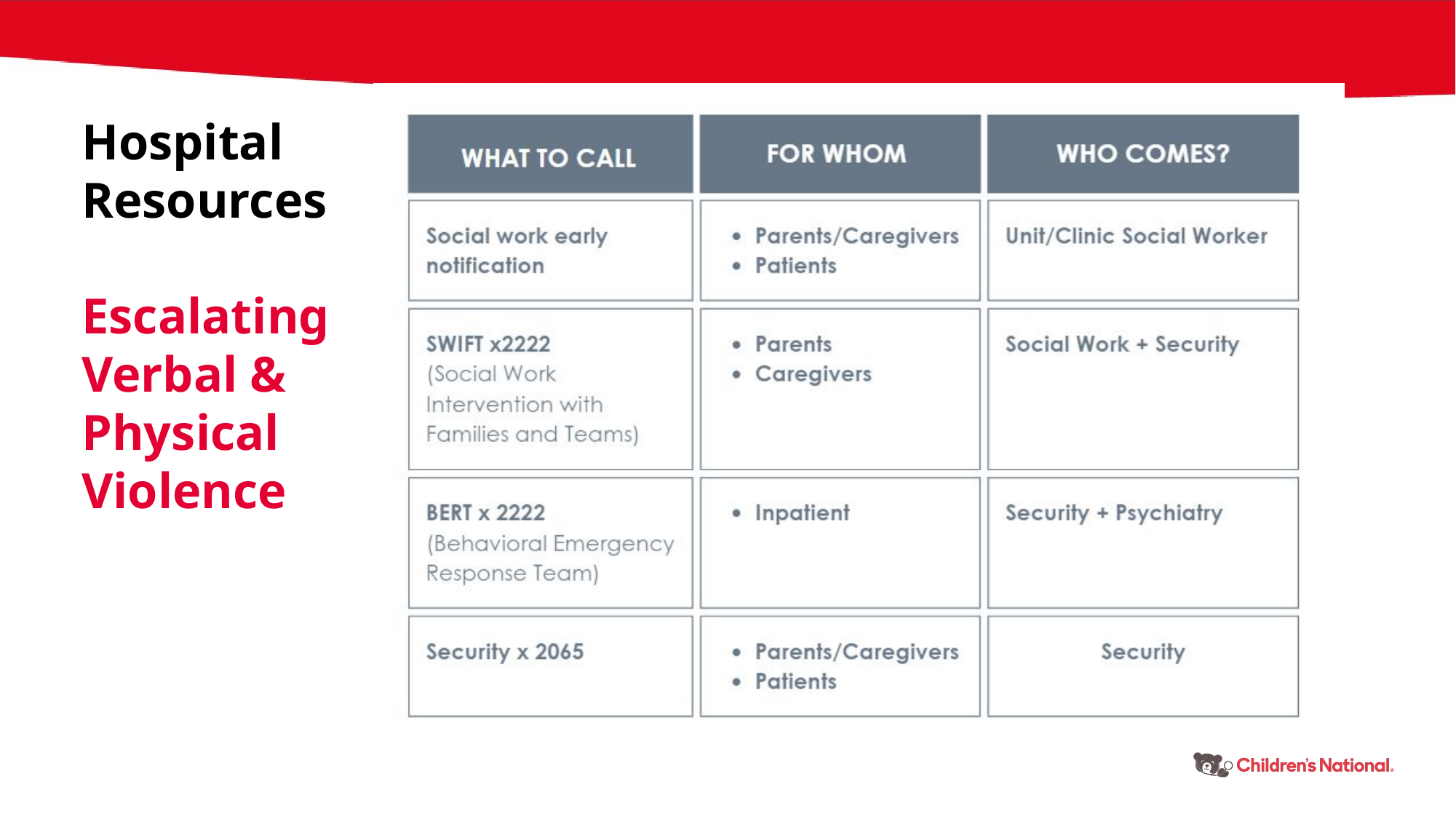

Hospital
Resources
Escalating
Verbal &
Physical
Violence

## Slide 12
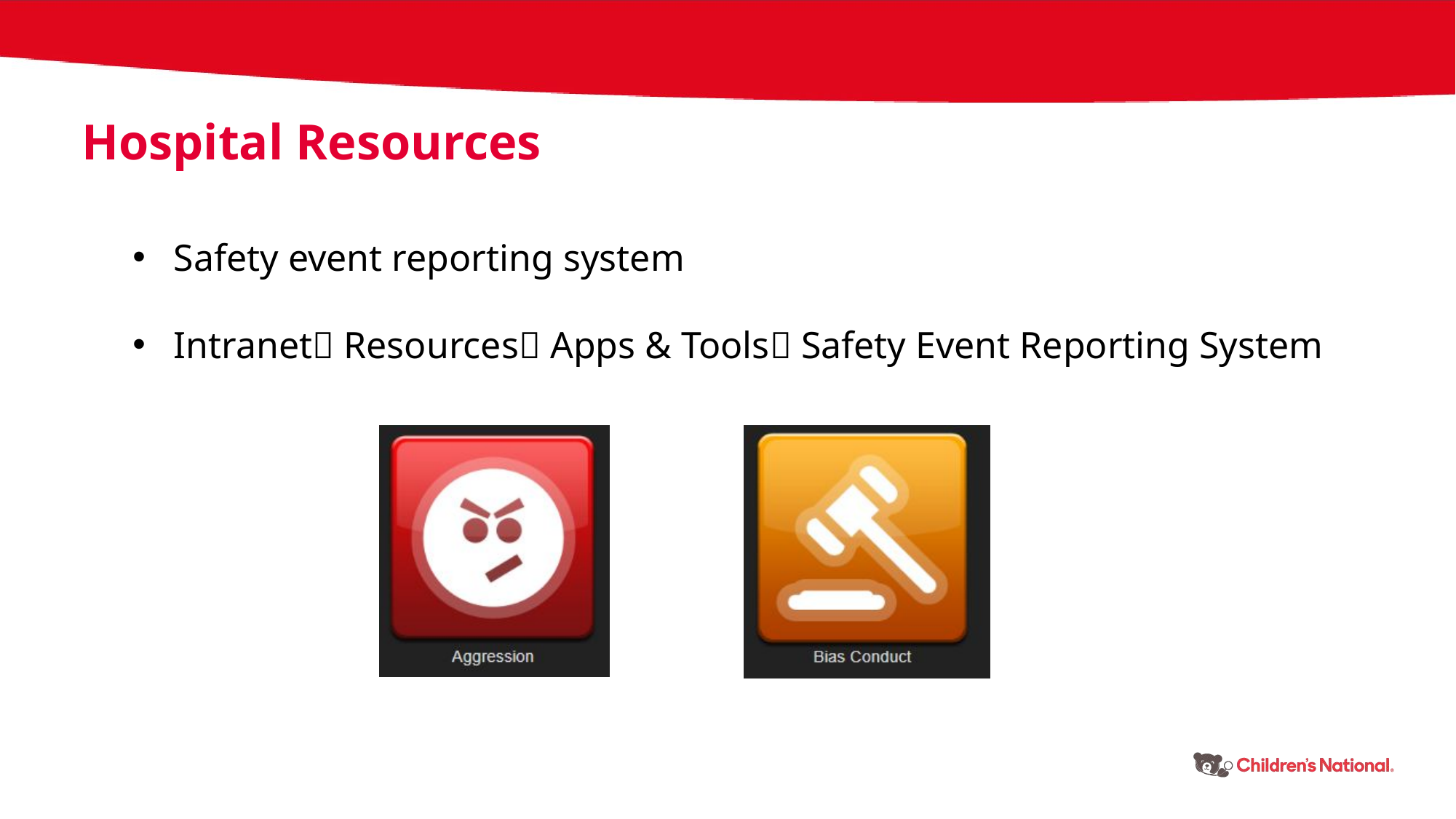

Hospital Resources
Safety event reporting system
Intranet Resources Apps & Tools Safety Event Reporting System

## Slide 13
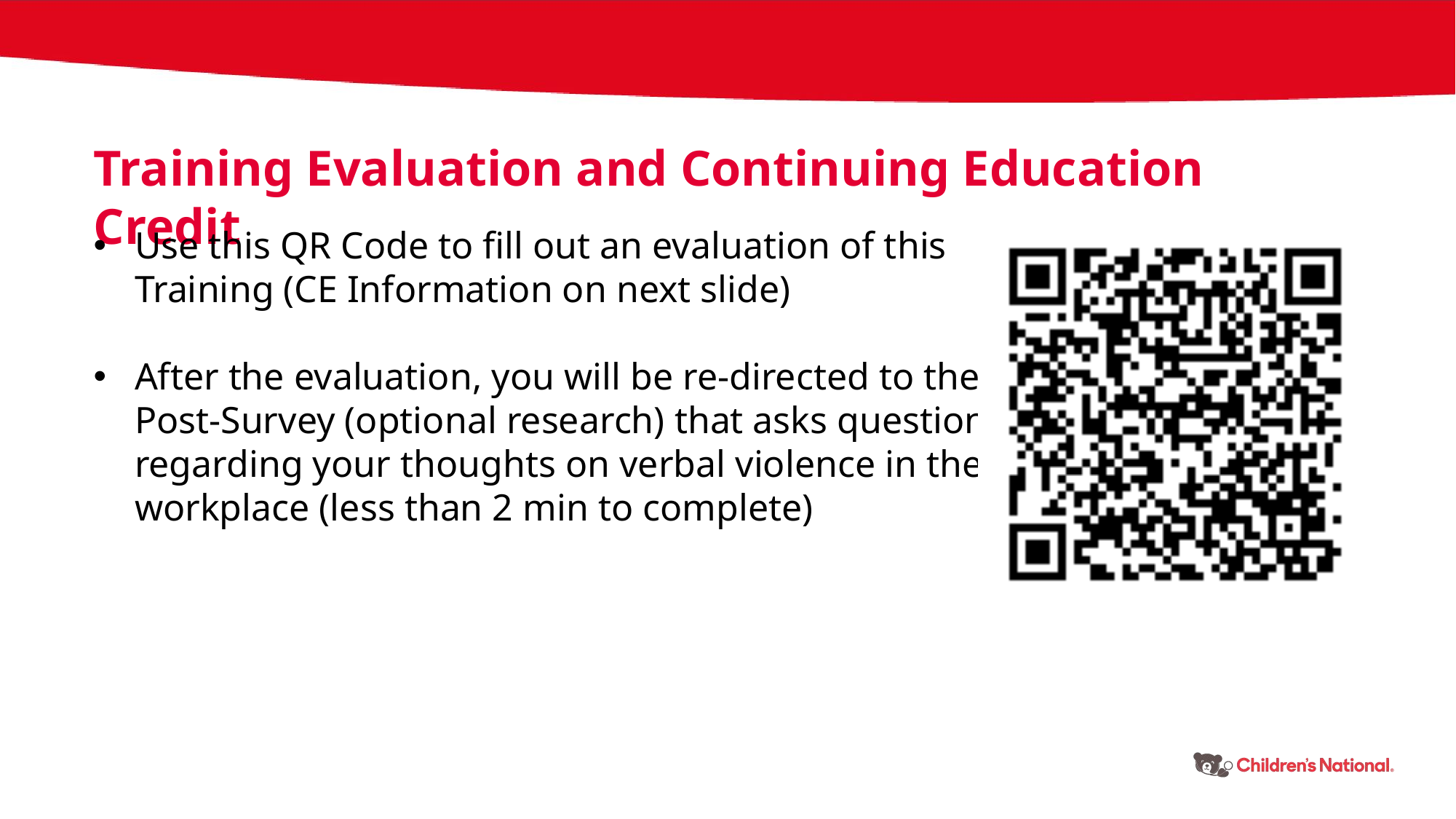

Training Evaluation and Continuing Education Credit
Use this QR Code to fill out an evaluation of this Training (CE Information on next slide)
After the evaluation, you will be re-directed to the Post-Survey (optional research) that asks questions regarding your thoughts on verbal violence in the workplace (less than 2 min to complete)

## Slide 14
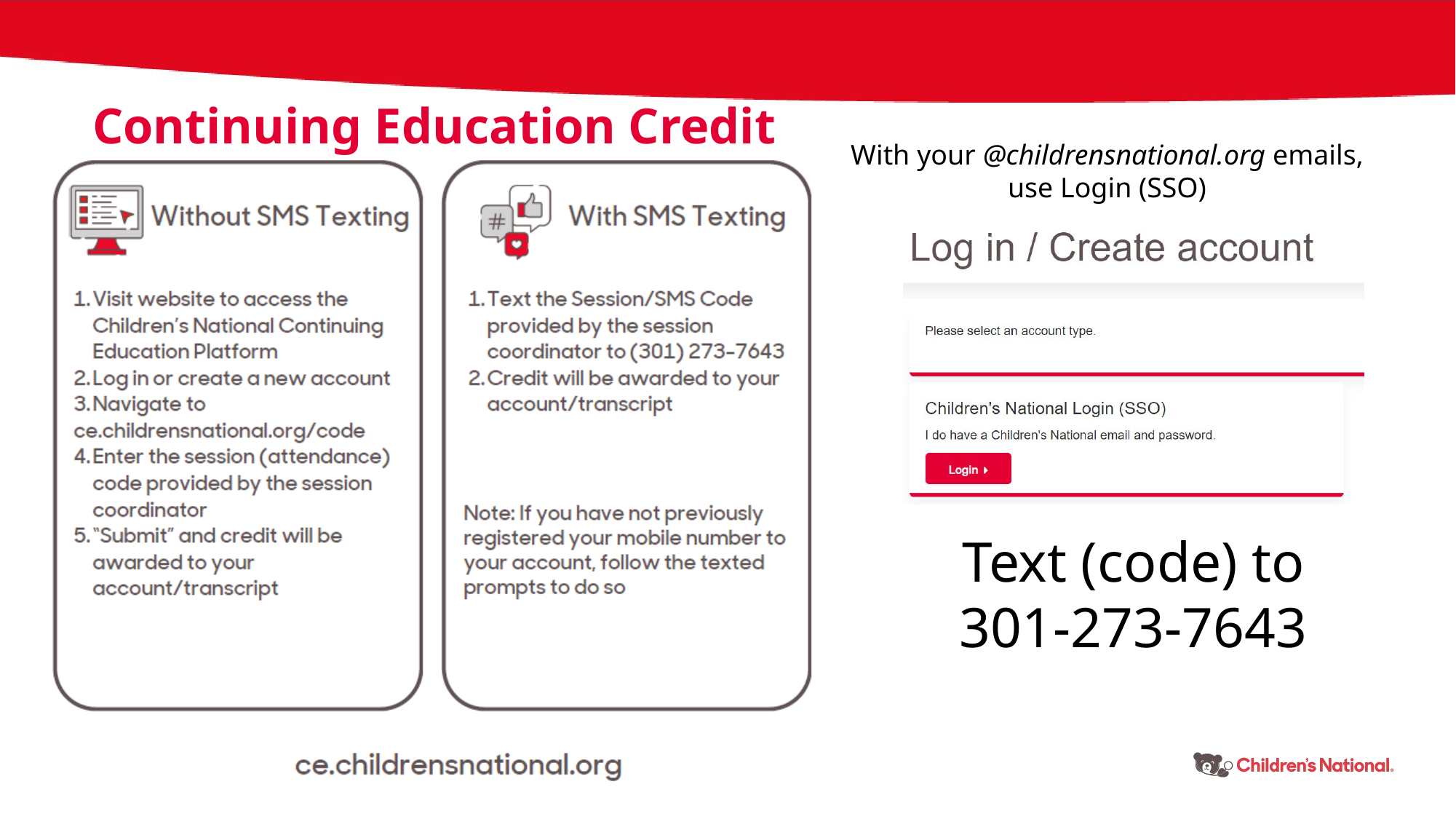

Continuing Education Credit
With your @childrensnational.org emails, use Login (SSO)
Text (code) to 301-273-7643

## Slide 15
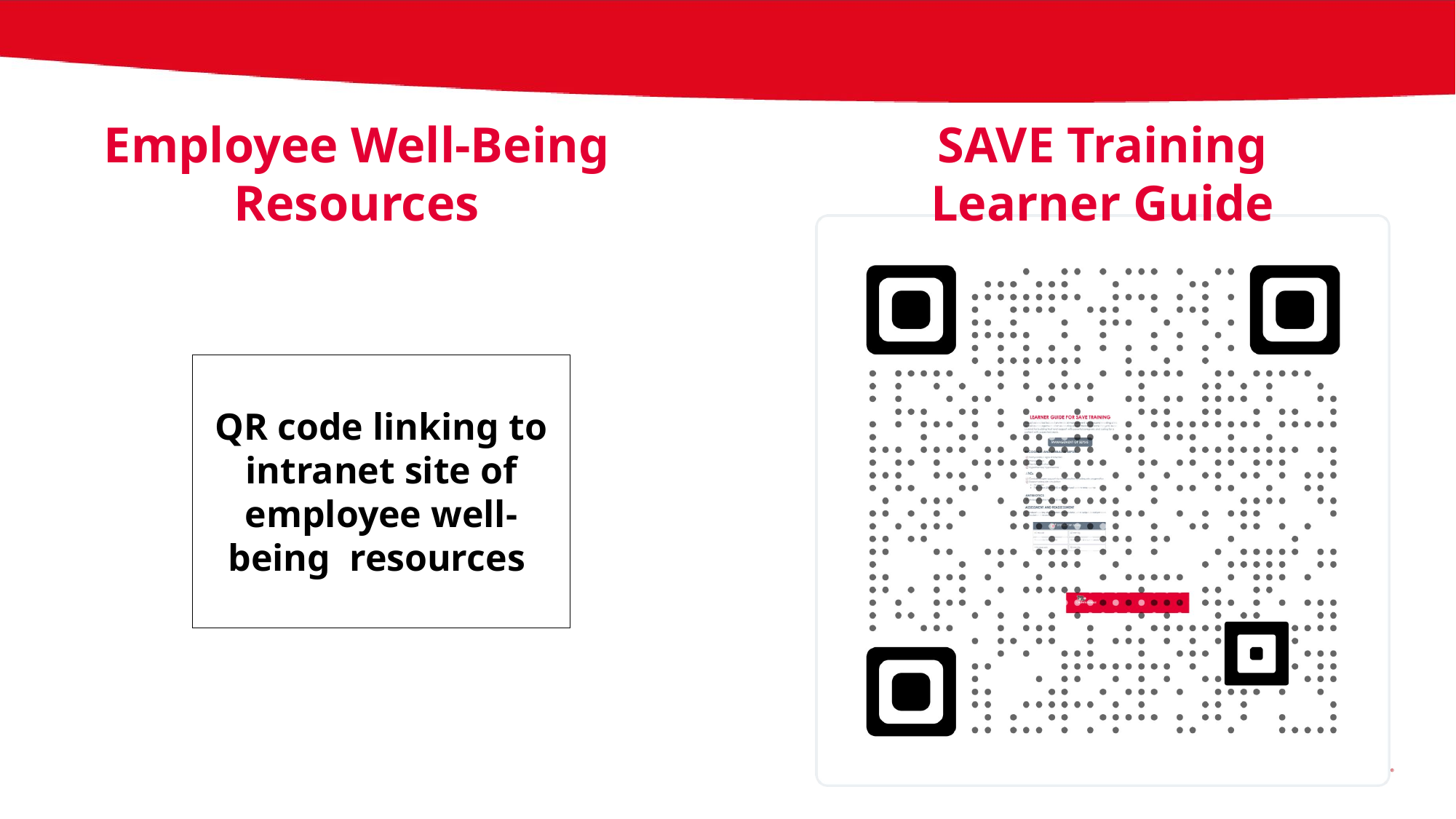

Employee Well-Being Resources
SAVE Training Learner Guide
QR code linking to intranet site of employee well-being resources

## Slide 16
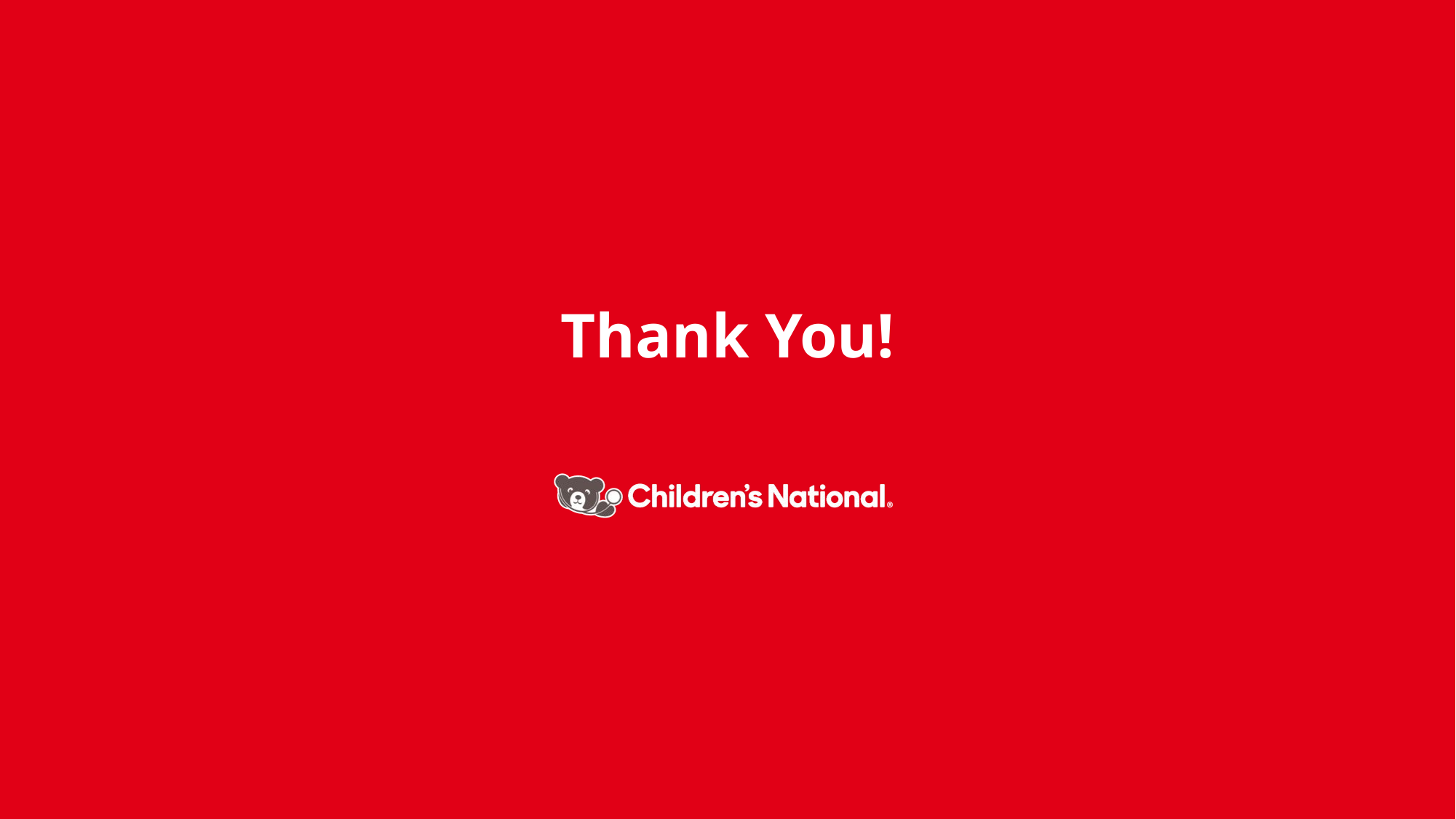

Supplement: Supplementary file 1 — Simulation Cases.docxSP Case.docxLearner Guide.pdfFacilitator Guide.docxTraining Slides.pptxTechnical Support Checklist.docxFlyer.pdfFeedback Survey.pdfFacilitator Debrief Worksheet.pdfPresurvey.pdf [file mep_2374-8265.11593-s001.zip › E. Training Slides.pptx]
