# Supplementary material for: Simulation Addressing Verbal Escalation (SAVE): An Interprofessional Simulation for Pediatric Health Care Professionals
Source: MedEdPORTAL. 2026 Apr 15;22:11593. doi: 10.15766/mep_2374-8265.11593 (PMC13080524; doi:10.15766/mep_2374-8265.11593)
Supplement: Supplementary file 1 — Simulation Cases.docxSP Case.docxLearner Guide.pdfFacilitator Guide.docxTraining Slides.pptxTechnical Support Checklist.docxFlyer.pdfFeedback Survey.pdfFacilitator Debrief Worksheet.pdfPresurvey.pdf [file mep_2374-8265.11593-s001.zip › F. Technical Support Checklist.docx]

**SAVE Training**

**Technical Support Checklist**

**Before session**

- Charge manikin: Toddler high-fidelity manikin with controller tablet and virtual monitor
- Charge laptop

***Supplies needed:***

- Patient crib
- Toddler high-fidelity monitors
- Laptop
- SAVE binder
- Code sheets for 2 scenarios
- Facilitator guides (3) for facilitators and sim ops specialist
- Sign in sheets for each session
- IRB information sheet
- Claiming continuing education information sheet
- SAVE facilitator debrief forms
- SAVE box
- ECG leads
- Pulse ox
- BP cuff
- Nasal cannula
- Nonrebreather mask
- Ambu bag and different sized masks
- IV attached to collection bag
- Labeled medications
- Normal saline, 1 L bag
- 20 cc syringe
- Stopcock
- Remote clicker for laptop
- IV pump and pole
- Bedsheet if in location other than Sim Center (for cart/bed)

**Session setup**

- Attach sim laptop to projector or TV (if in Sim Center) with slides loaded
- Change code on continuing ed slide (in sheet protector at front of SAVE binder; new code for each day of sessions)
- Insert dongle from clicker into side of laptop
- Set up manikin and supplies on table
- Assist learners with signing in for session (will typically be outside of classroom with forms on bedside table)
- Name tags and sharpie marker
- Sign-in sheets
- Study information sheet
- Tape pre-survey on wall outside of room and ask participants to complete if they haven’t already

**Scenario 1**

- Run manikin VS for scenario 1
- Reset equipment during debrief 1
- Change code sheet

**Scenario 2**

- Run manikin for scenario 2
- Reset equipment during debrief of scenario 2 for next session during debrief 2
- Change code sheet
- Assist learners signing in for next session
